# From Screening to Site Control: Phytic-Acid Mediated P-Tuning of M–N Coordination to Balance Iodine Adsorption and Stability in Zn–I_2_ Batteries

**DOI:** 10.1007/s40820-026-02321-6

**Published:** 2026-07-30

**Authors:** Yuxuan Jiang, Bingxin Sun, Mohsen Shakouri, Bin He, Wang Zhang, Ran Wang, Tianxiao Sun, Huan Pang

**Affiliations:** 1 School of Chemistry and Materials, Yangzhou University, Yangzhou, 225002 People’s Republic of China; 2 Canadian Light Source, University of Saskatchewan, Saskatoon, SK S7N 2V3 Canada; 3 Department of Materials Engineering, Zhejiang Key Laboratory for Industrial Solid Waste Thermal Hydrolysis Technology and Intelligent Equipment, Huzhou Normal University, Huzhou, 313000 People’s Republic of China; 4 Yangzhou Key Laboratory of Smart Materials and Clean Energy, Interdisciplinary Research Center for Advanced Energy, Yangzhou University, Yangzhou, 225002 People’s Republic of China; 5 Shanghai Synchrotron Radiation Facility, Shanghai Advanced, Research Institute, Chinese Academy of Sciences, Shanghai, 201204 People’s Republic of China

**Keywords:** Zinc-iodine batteries, Single-atom catalyst, Moderate adsorption, Heteroatom doping, Core–shell structure

## Abstract

Aqueous zinc-iodine batteries (AZIBs) show promise for grid-scale energy storage, but they are hampered by polyiodide shuttling, sluggish iodine redox kinetics, and irreversible active-site poisoning caused by uncontrolled adsorption. We provide a comprehensive screening of M_1_ (M_1_ = P, S, B) heteroatom dopants, and P is identified as the best candidate for achieving coordination-tuned, moderate adsorption that balances adsorption and catalytic activity while mitigating site poisoning. Using phytic acid as both the P source and an etchant, we create a universal in situ approach to core–shell single-atom catalysts (M_2_-P-CSNC, M_2_ = Fe, Co, Ni). The unique core–shell structure achieves stable confinement of polyiodides, rapid ion transport, and protection of active sites, while in situ P doping precisely regulates the local electronic environment and *d*-band center of the Fe–N_*x*_ active centers. In situ characterization confirms that Fe–P-CSNC has a strong reversible anchoring ability for polyiodides, which can significantly accelerate redox kinetics. The optimized Fe–P-CSNC/I_2_ exhibits almost no capacity decay after 20,000 cycles at a current density of 2 A g^−1^. This work’s facile heteroatom doping strategy for electronic modulation offers a reference for high-performance catalyst design in conversion-type energy storage systems.Kindly check and confirm the edit made in the title.1. We have checked and confirmed the edited title. 2. We found some issues with Figure 3d and have uploaded the revised image as an attachment.

**Supplementary Information:**

The online version contains supplementary material available at 10.1007/s40820-026-02321-6.

## Highlights

- Systematic B/S/P heteroatom screening identifies P doping as the optimal strategy to realize coordination-tuned moderate iodine adsorption, addressing the critical adsorption-catalysis imbalance in Zn-I_2_ batteries.
- A universal phytic acid-assisted one-step strategy is developed to fabricate core-shell single-atom catalysts, with synchronous P doping, cavity construction and metal site atomic dispersion.

**Supplementary Information:**

The online version contains supplementary material available at 10.1007/s40820-026-02321-6.

## Graphical abstract

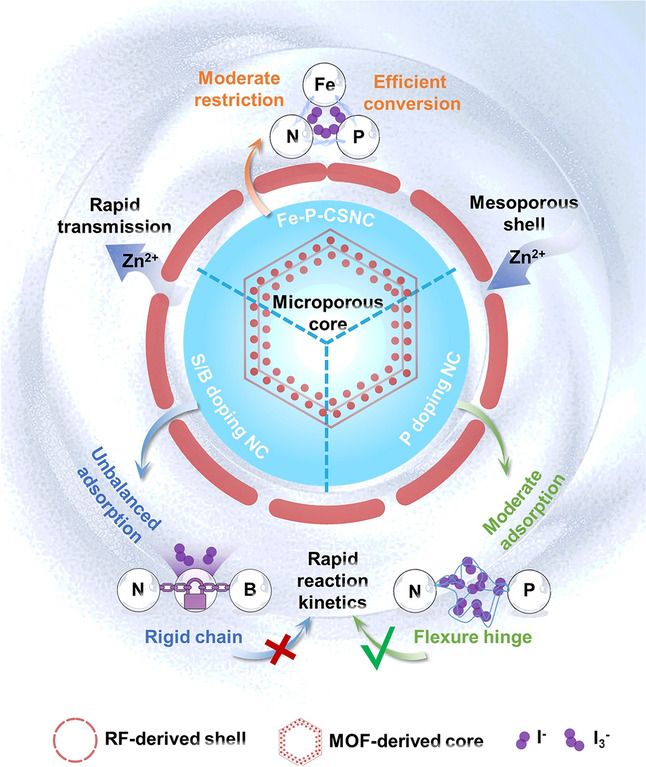

## Introduction

AZIBs have emerged as compelling candidates for next-generation grid-scale energy storage systems, owing to their inherent merits of low cost, intrinsic operational safety, a high theoretical iodine capacity of 211 mAh g^−1^, and environmental benignity [1–3]. Nevertheless, the practical deployment of AZIBs is severely hindered by two interlinked, long-standing bottlenecks: the notorious shuttle effect driven by soluble polyiodide intermediates (predominantly I_3_^−^) at the iodine cathode and the intrinsically sluggish redox kinetics of the I^−^/I_2_ couple. The former triggers irreversible loss of active iodine species, progressive corrosion and passivation of the zinc anode, and continuous degradation of coulombic efficiency over prolonged cycling [4, 5].

Conventional carbonaceous hosts, including porous carbon, carbon nanotubes, and graphene, can physically confine iodine species via porous architectures [6–9], yet their nonpolar surfaces afford only weak interactions with polar polyiodides, failing to effectively suppress the shuttle effect upon repeated cycling [10–12]. ZIF-8-derived porous carbons, featuring ultrahigh specific surface area, hierarchical porosity and tunable single-heteroatom doping (e.g., B, S, P) [13, 14], enable enhanced iodine confinement through the combination of physical pore entrapment and polar chemisorption sites. However, most related studies exclusively pursue maximized adsorption energy toward polyiodides, while completely overlooking the detrimental impact of excessive adsorption on the desorption of oxidation products during charging. Such unbalanced adsorption–desorption behavior inevitably induces irreversible passivation of active sites and severely retarded oxidation kinetics, a hidden root cause of rapid capacity fade. To date, systematic investigations into the rational screening of heteroatoms to achieve balanced adsorption and reversible conversion of iodine intermediates remain scarce.

Single-atom catalysts (SACs) have emerged as a promising platform to address these kinetic limitations, with well-defined active centers, tailorable electronic structures, and maximum atomic utilization, demonstrating remarkable efficacy in accelerating the conversion kinetics of iodine intermediates. Conventional metal-N_*x*_ sites, however, suffer from two critical drawbacks: unbalanced adsorption of iodine species and insufficient bidirectional catalytic activity arising from mismatched electronic distribution, as well as severe site poisoning and passivation caused by direct exposure of active centers to the electrolyte [15–18]. It is well-established that the adsorption strength of reaction intermediates on metal centers is strongly correlated with the position of the d-band center relative to the Fermi level [18, 19]. To this end, we propose a synergistic strategy integrating hierarchical core–shell structure engineering and heteroatom doping, which enables anti-poisoning protection of active sites via spatial confinement, as well as precise modulation of the local electronic environment and d-band center of metal-N_*x*_ centers, ultimately achieving balanced adsorption–desorption and reversible redox cycling of iodine intermediates [20].

Herein, we first adopt a facile post-doping strategy to rapidly screen ZIF-8-derived carbon hosts doped with B, S, and P single heteroatoms, and identify P doping as the optimal heteroatom to balance polyiodide adsorption and iodine species desorption for stable reversible redox cycling in AZIBs. Nevertheless, the traditional post-doping method suffers from intrinsic drawbacks, including inhomogeneous heteroatom distribution and inability to synchronously construct a hierarchical core–shell cavity structure for polyiodide confinement. Building on the screening result, we develop a facile, universal phytic acid-assisted in situ synthetic strategy using ZIF-8 as the precursor, which perfectly addresses the limitations of post-doping. Leveraging the dual role of phytic acid as both the phosphorus source and in situ etching agent, we achieve in situ uniform P doping, hierarchical core–shell cavity structure construction, and atomic dispersion of transition metal centers in a one-pot step. The hierarchical core–shell architecture acts as a dedicated nanoreactor for iodine species [21, 22], integrating sufficient iodine loading capacity, robust polyiodide confinement, rapid ion transport, and full exposure of active sites. Meanwhile, the vicinal P doping precisely modulates the local electronic properties and d-band center position of Fe–N_*x*_ active centers, realizing balanced adsorption–desorption of iodine species.

The structure-performance relationship and reaction mechanism are systematically revealed via multi-scale electrochemical characterizations, theoretical calculations, and in situ monitoring techniques. The optimized Fe–P-CSNC/I_2_ cathode delivers a high discharge specific capacity of 146 mAh g^−1^ at 5 A g^−1^, exceptional rate capability, and ultra-long cycling stability with negligible capacity decay after 20,000 cycles at 2 A g^−1^. The moderate adsorption design principle and the facile in situ heteroatom doping strategy developed herein provide a rational guideline for addressing the intermediate shuttle effect and unbalanced bidirectional redox kinetics in various conversion-type energy storage systems.

## Experimental Section

### Materials

Fe(NO_3_)_3_·9H_2_O, Co(NO_3_)_2_·6H_2_O, Ni(NO_3_)_2_·6H_2_O, anhydrous ethanol, methanol, Boric acid, thiourea, anhydrous sodium hypophosphite, formaldehyde and resorcinol were obtained from Aladdin (China). NH_3_·H_2_O, Zn(NO_3_)_2_·6H_2_O, phytic acid, Super P and N-Methyl pyrrolidone (NMP) were purchased from Shanghai Chemical Reagents Company (Shanghai, China). All the reagents used are analytically pure.

### Materials Synthesis

#### Synthesis of ZIF-8

ZIF-8 were prepared by one-pot coprecipitation procedure. Briefly, 2-MeIm (11.3 g) was first dissolved in deionized water (164 mL) to form a clear aqueous solution. Then, 36 mL of aqueous solution containing Zn (NO_3_)_2_·6H_2_O (725 mg) was rapidly added to above solution and stirred for 24 h to form ZIF-8. Finally, the ZIF-8 was centrifuged at 10,000 rpm, and the obtained white-colored Zn-MOF precipitate was washed with ethanol several times and finally dried at 60 °C for 12 h.

#### Preparation of ZIF-8@RF

Add the ZIF-8(100 mg) to a solution containing ethanol (3 mL), H_2_O (7 mL), and NH_3_·H_2_O (0.5 mL) at room temperature with stirring. After stirring for 30 min, add resorcinol (0.175 g) and formaldehyde (0.3 mL) to the solution, stir for 24 h, centrifuge the precipitate at a rate of 6000 r min^−1^, wash with water and ethanol, and dry at 60 °C for about 12 h to obtain the precipitate ZIF-8@RF composite materials.

#### Preparation of NC

The dried ZIF-8@RF composites were heated from room temperature to 920 °C in a tube furnace under nitrogen atmosphere at an increasing rate of 2 °C min^−1^ for 2 h under nitrogen atmosphere and then cooled to room temperature. The obtained samples were named as NC.

#### ***Preparation of M***_***1***_***-NC (M***_***1***_ = ***P, S, B)***

100 mg of NC and 100 mg of boric acid/500 mg of thiourea/1 g of anhydrous sodium hypophosphite were placed in the downstream and upstream sections of a tube furnace, respectively. The samples were heated to 600 °C at a rate of 2 °C min^−1^ in a nitrogen atmosphere and held at that temperature for 3 h, thereby producing M_1_-NC.

#### ***Preparation of M***_***2***_***-ZIF-8@PA@RF (M***_***2***_ = ***Fe, Co, Ni)***

Add 24.4 μmol Fe(NO_3_)_3_·9H_2_O/Co(NO_3_)_2_·6H_2_O/Ni(NO_3_)_2_·6H_2_O during the synthesis process of ZIF-8 to obtain M_2_-ZIF-8. Disperse 300 mg M_2_-ZIF-8 in 100 mL of methanol, add 150 μL phytic acid, stir for 20 min, and then centrifuge. Directly disperse the centrifuged product in a solution containing ethanol (3 mL), H_2_O (7 mL), and NH_3_·H_2_O (0.5 mL) at room temperature with stirring. After stirring for 30 min, add resorcinol (0.175 g) and formaldehyde (0.3 mL) to the solution, stir for 24 h, centrifuge the precipitate at a rate of 600 r min^−1^, wash with water and ethanol, and dry at 60 °C for about 12 h to obtain the precipitate M_2_-ZIF-8@PA@RF composite materials.

#### ***Preparation of M***_***2***_***-P-CSNC***

The dried M_2_-ZIF-8@PA@RF composites were heated from room temperature to 920 °C in a tube furnace at an increasing rate of 2 °C min^−1^ for 2 h under nitrogen atmosphere. The obtained samples were named as M_2_-P-CSNC.

### Preparation of Cathode

M_1_-NC and M_2_-P-CSNC were used as the activated material of Zn-iodine cells through melt-diffusion method (samples and iodine mixed uniformly into the hydrothermal reaction kettle sealed, warmed to 120 °C insulation 12 h). For the preparation of cathode, samples/I_2_, Super P, and NMP solution containing 5 wt% PVDF (8:1:1 by weight) were milled and stirred to form uniform slurry. Then, the slurry was coated on the carbon paper and dried in vacuum at 60 °C for 12 h.

## Results and Discussion

### Material Synthesis and Structural Characterizations

To rapidly screen the optimal heteroatom for balancing iodine species adsorption and desorption, we first prepared M_1_-NC via a facile post-doping method, which enables rapid batch preparation of heteroatom-doped carbon samples for primary performance screening. Based on the preliminary screening result that P doping delivers the most balanced electrochemical performance, we further developed a phytic acid-assisted in situ one-pot strategy to fabricate M_2_-P-CSNC, addressing the intrinsic limitations of the post-doping method.

**Fig. 1:**
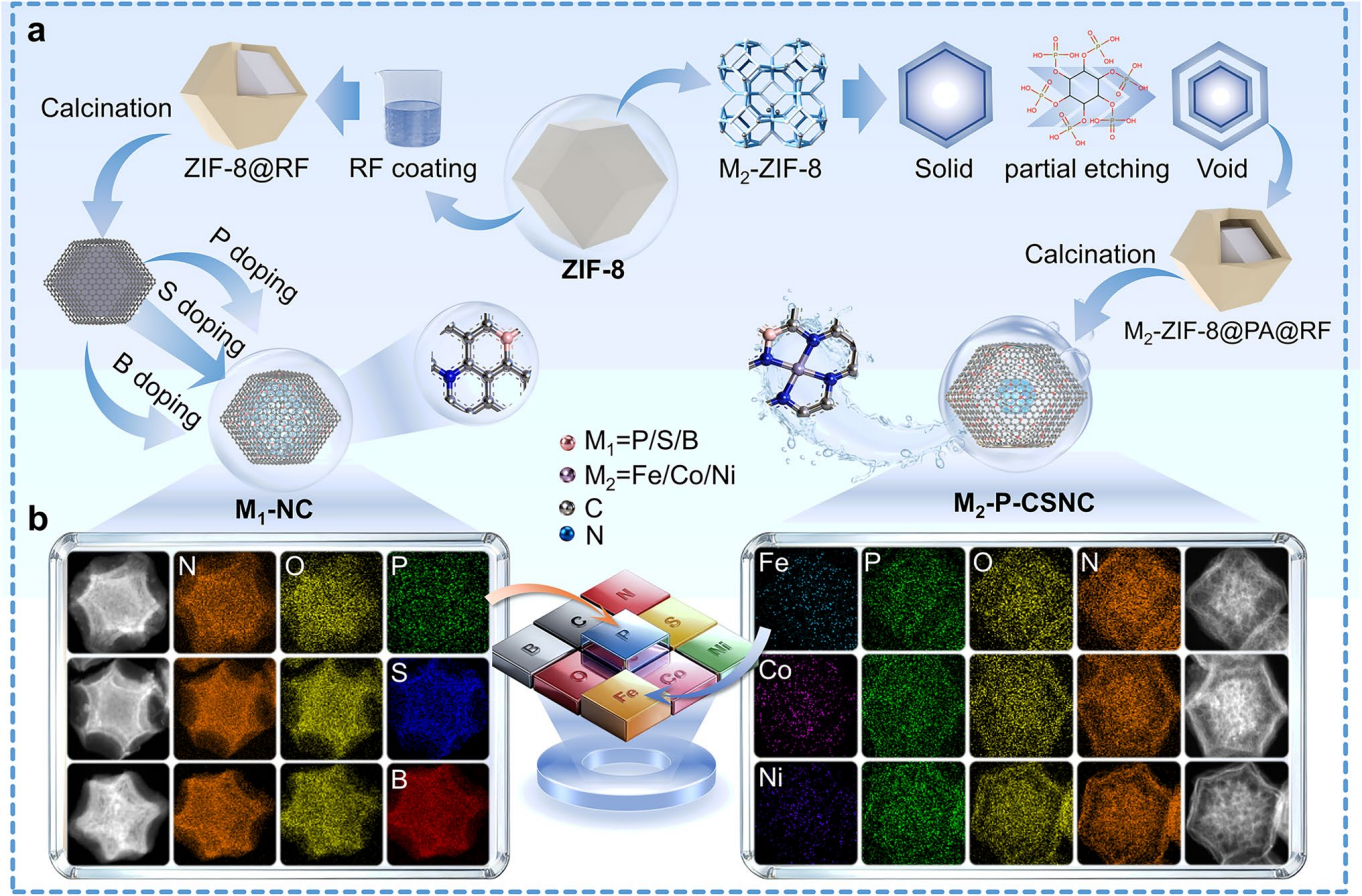
Design and characterization of various iodine hosts: **a** synthesis roadmap and **b** Energy-Dispersive Spectrometer (EDS) elemental mapping

Figure 1a illustrates the synthesis pathways. ZIF-8 is first coated with resorcinol–formaldehyde resin (RF) to generate ZIF-8@RF, which is then carbonized at high temperature and subjected to a subsequent heat treatment to add P, S, or B. The core–shell structure M_2_-P-CSNC is manufactured as follows: M_2_-doped ZIF-8 precursors (M_2_-ZIF-8) are produced using a straightforward solvothermal approach. M_2_ partially replaces Zn^2+^ in the framework, resulting in homogenous M-N_*x*_ coordination sites. Phytic acid is then used for in-situ etching, with its phosphate groups preferentially coordinating surface metal ions while gradually etching the ZIF-8 core and preserving the external framework, resulting in a tunable interior cavity and uniform anchoring of P. The etched precursors are then coated with RF and pyrolyzed to produce M_2_-P-CSNC, which has a well-defined interior cavity and uniform P inclusion.

EDS elemental mapping is displayed in Fig. 1b. After high-temperature calcination, all samples kept the distinctive framework structure of the ZIF-8 precursor, with no discernible structural collapse or particle aggregation, thanks to the confinement protective effect of the RF shell. The P, S, and B as well as the C, N, and O matrix elements were distributed uniformly throughout the whole structure for the M_1_-NC. The M_2_-P-CSNC, which were made by phytic acid in-situ etching, had a distinctive internal cavity and a unique core–shell structure. The Fe, Co, and Ni metal active sites were evenly distributed throughout the carbon matrix without any visible metal agglomeration, offering plenty of accessible active sites for the iodine redox reaction [23].

**Fig. 2:**
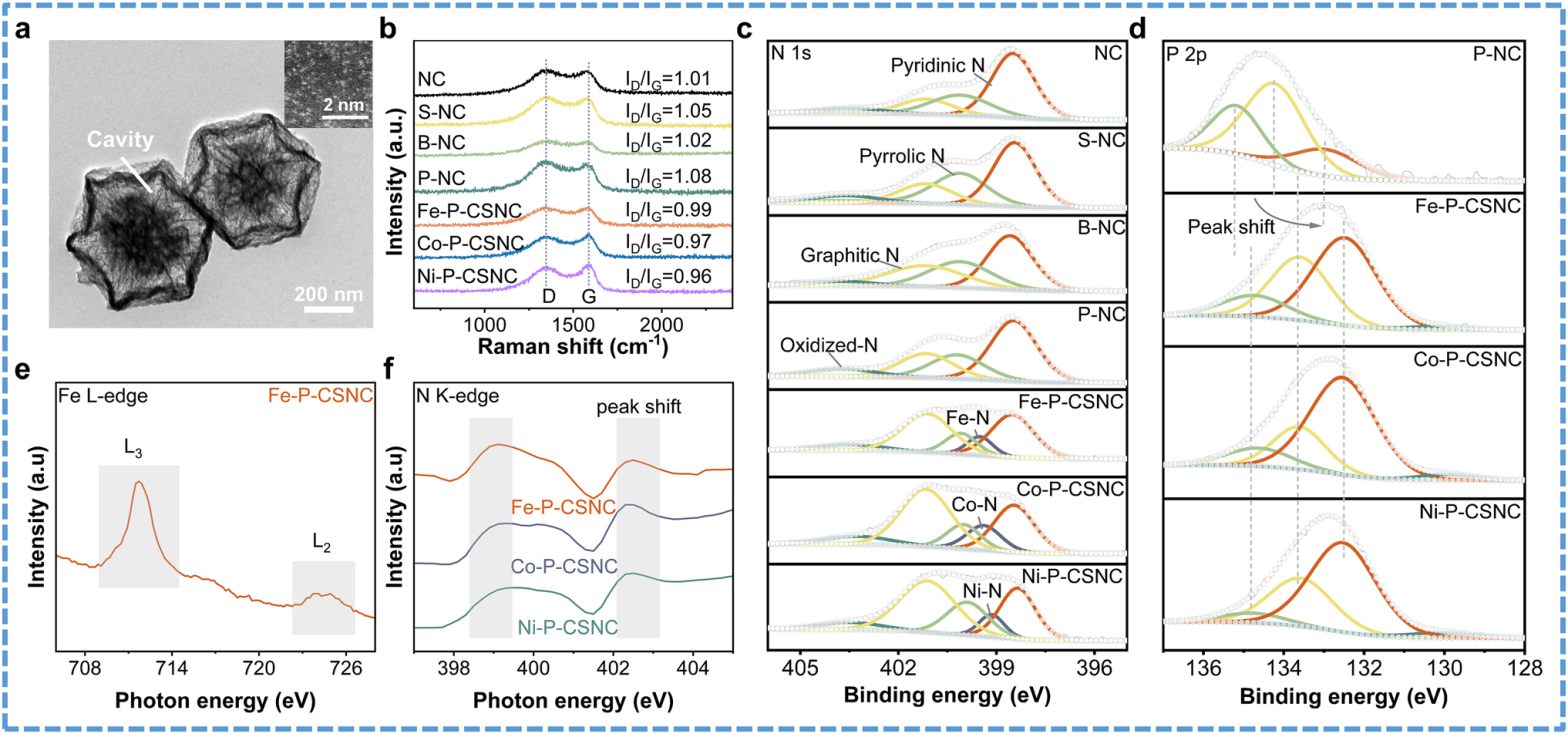
**a** TEM and HAADF-STEM images of Fe–P-CSNC. **b** Raman spectroscopy. XPS spectra of **c** N 1*s* and **d** P 2*p*. XANES spectra for **e** Fe–P-CSNC and **f** M_2_-P-CSNC

Scanning electron microscopy (SEM) and transmission electron microscopy (TEM) were used to examine the materials’ morphological and structural features. The samples are all about 500 nm in size and have a dodecahedron-like form (Figs. S1, S2, and 2a). The homogeneous distribution of active sites in M_2_-P-CSNC, as seen in Fig. 1b, is confirmed by the high-angle annular dark field scanning transmission electron microscopy (HAADF-STEM) image (Fig. 2a inset). Additionally, the large specific surface area of M_2_-P-CSNC and its distinctive structure with mesoporous and microporous hierarchical pores are displayed in Fig. S3. In addition to maintaining an adequate specific surface area to support a high surface loading of iodine, the core–shell-cavity hierarchical pore structure offers practical electrolyte channels and short diffusion paths, which lower the mass transfer resistance of Zn^2+^ in the cathode region and increase the interfacial reaction rate [24–26].

The crystal structure of the catalysts was analyzed by X-ray diffraction (XRD) and Raman spectroscopy. All samples exhibit a broad peak at around 23°, which corresponds to the (002) crystal plane of amorphous carbon. Compared with NC, the diffraction peaks of M_1_-NC show slight shifts in different degrees (Fig. S4) [27]. This phenomenon originates from the lattice distortion and interlayer spacing change in the carbon framework caused by the incorporation of heteroatoms. For the M_2_-P-CSNC, no sharp characteristic peaks corresponding to metals are observed in the XRD patterns, indicating that the metal species do not form nano-scale aggregates and are uniformly dispersed in the carbon matrix at the atomic level. This result is fully consistent with the previous EDS elemental mapping (Fig. 1b) and HAADF-STEM (Fig. 2a). Inductively coupled plasma mass spectrometry (ICP-MS) revealed Fe loadings of 1.96 wt% for Fe–P-CSNC (Table S1), further confirming that our phytic acid-assisted strategy enables efficient atomic-level loading of Fe active sites without obvious metal agglomeration. To elucidate the differences in chemical states and electronic structures between M_1_-NC and M_2_-P-CSNC, as well as their structure-performance relationship in AZIBs, X-ray photoelectron spectroscopy (XPS) and synchrotron radiation-based soft X-ray absorption near-edge structure (XANES) characterizations were performed on the as-prepared samples. Figure S5 demonstrates that all samples contain C, N, and O. The Raman spectra of each sample are displayed in Fig. 2b. The graphitized structure of *sp*^2^-hybridized carbon is shown by the *G* peak at 1580 cm^−1^, while the flaws and disordered structure of carbon materials are represented by the *D* peak at 1350 cm^−1^. The I_D_/I_G_ ratio is somewhat raised by single heteroatom doping, which introduces a few flaws. The high-temperature catalytic graphitization effect of transition metals causes the I_D_/I_G_ ratio to decrease after adding metal single atoms, preserving the materials’ strong electrical conductivity. The high-resolution N 1*s* XPS spectra of M_1_-NC and M_2_-P-CSNC are presented in Fig. 2c. For NC, the N 1*s* spectrum can be deconvoluted into four characteristic peaks located at ~ 398.6, ~ 399.8, ~ 401.1, and ~ 402.5 eV, corresponding to pyridinic N, pyrrolic N, graphitic N, and oxidized N species [25]. With the introduction of S, B, and P, the main peaks of M_1_-NC samples gradually shift toward higher binding energy, indicating a progressive increase in the relative proportion of graphitic N and pyrrolic N. In comparison, an additional characteristic peak emerges at ~ 399.2 eV in the N 1*s* spectra of M_2_-P-CSNC, which is ascribed to M-N_*x*_ coordination bonds. This verifies the formation of atomically dispersed metal sites anchored by N atoms within the carbon matrix. Meanwhile, the main peaks of M_2_-P-CSNC shift further toward higher binding energy, which can be attributed to the coordination of free pyridinic N with metal centers to form M-N_*x*_ active sites, reducing the proportion of uncoordinated free pyridinic N. The increased proportion of graphitic N can significantly enhance the electronic conductivity of the carbon matrix, accelerate electron transfer during the iodine redox process, and serve as adsorption sites for polyiodides, which synergistically suppresses the shuttle effect together with M-N_*x*_ active sites.

Figure 2d shows the high-resolution P 2*p* XPS spectra of P-NC and M_2_-P-CSNC series samples. No characteristic peaks corresponding to metal-phosphorus bonds are observed in the spectra, indicating that P introduced via phytic acid does not participate in the formation of metal phosphides. For the P-NC, its P 2*p* spectrum can be deconvoluted into the P–C and P–O, verifying the successful incorporation of P heteroatoms into the carbon framework. Compared with P-NC, the characteristic peaks of all M_2_-P-CSNC samples shift toward lower binding energy, among which Fe–P-CSNC shows the most significant shift, confirming the strongest electronic interaction and electron transfer effect between P and Fe centers. Rather than regulating the properties of active sites via the formation of stable metal-phosphorus bonds, P species are in the vicinity of the metal centers. They modulate the electronic structure of M-N_*x*_ sites through the vicinal electron push–pull effect and local geometric distortion, break the planar symmetry of the pristine structure, and induce slight coordination asymmetry, thereby precisely tuning the d-band center of the metal centers. Compared with Co and Ni, the 3*d* electron configuration and orbital energy level of Fe are more readily tuned to the optimal range through the synergistic effect of vicinal P and N, endowing Fe sites with a moderate adsorption strength toward I_3_^−^/I_2_ species. This moderate adsorption strength can not only effectively inhibit the shuttle effect of polyiodides, but also avoid the irreversible passivation of active sites caused by excessively strong adsorption, achieving the critical balance between polyiodide confinement and catalytic reversibility required for high-performance Zn-I_2_ batteries.

To further reveal the electronic structure, oxidation state, and intrinsic catalytic activity of Fe species in Fe–P-CSNC, soft XANES characterization at the Fe *L*-edge was performed, with the results shown in Fig. 2e. The spectrum exhibits two distinct characteristic absorption peaks at ~ 711 and ~ 725 eV, corresponding to the *L*_3_-edge and *L*_2_-edge of Fe. This result indicates that Fe maintains a coordinated oxidized structure with partially unoccupied 3*d* states, which confirms that Fe in Fe–P-CSNC exists in a positively charged oxidized state. High-resolution XPS Fe 2*p* spectra (Fig. S6) further verifies the mixed Fe^2+^/Fe^3+^ positive oxidation state of Fe species, consistent with the XANES findings. Figure 2f shows the N *K*-edge of M_2_-P-CSNC. All spectra exhibit two dominant absorption peaks in the range of 398–404 eV. Among them, the peak at ~ 399.5 eV is ascribed to the electronic transition from the N 1*s* orbital to the π* orbital of pyridinic N (especially M-N_*x*_ species), and the peak at ~ 401.8 eV corresponds to the electronic transition associated with graphitic N species. Strikingly, compared with Co-P-CSNC and Ni–P-CSNC, the characteristic peaks of Fe–P-CSNC show a significant shift toward higher photon energy. This phenomenon indicates that the electron density around N atoms undergoes a remarkable redistribution upon P doping, a stronger electron transfer from N atoms to Fe centers occurs. This result further confirms the strong perturbation effect of P on the Fe–N coordination environment.

The robust Fe–N coordination bonds can greatly improve the structural stability of the active sites, effectively inhibit the agglomeration and dissolution of metal sites during long-term charge–discharge cycles, and ensure the sustained exposure of active sites and stable catalytic activity throughout long cycling [28]. Second, the continuous electron transfer from N to Fe can further optimize the electronic structure of Fe sites and continuously enhance its electrocatalytic activity and kinetic acceleration capability for the iodine redox reaction. In contrast, the electronic interaction of Co–N and Ni–N bonds is weaker, and the perturbation effect of P doping on their coordination environment is limited. The structural stability of active sites and the optimization effect of electronic structure are inferior to those of Fe–P-CSNC; thus, they are more prone to activity decay during long-term cycling.

### Electrochemical Performance of Zn-I_2_Batteries

**Fig. 3:**
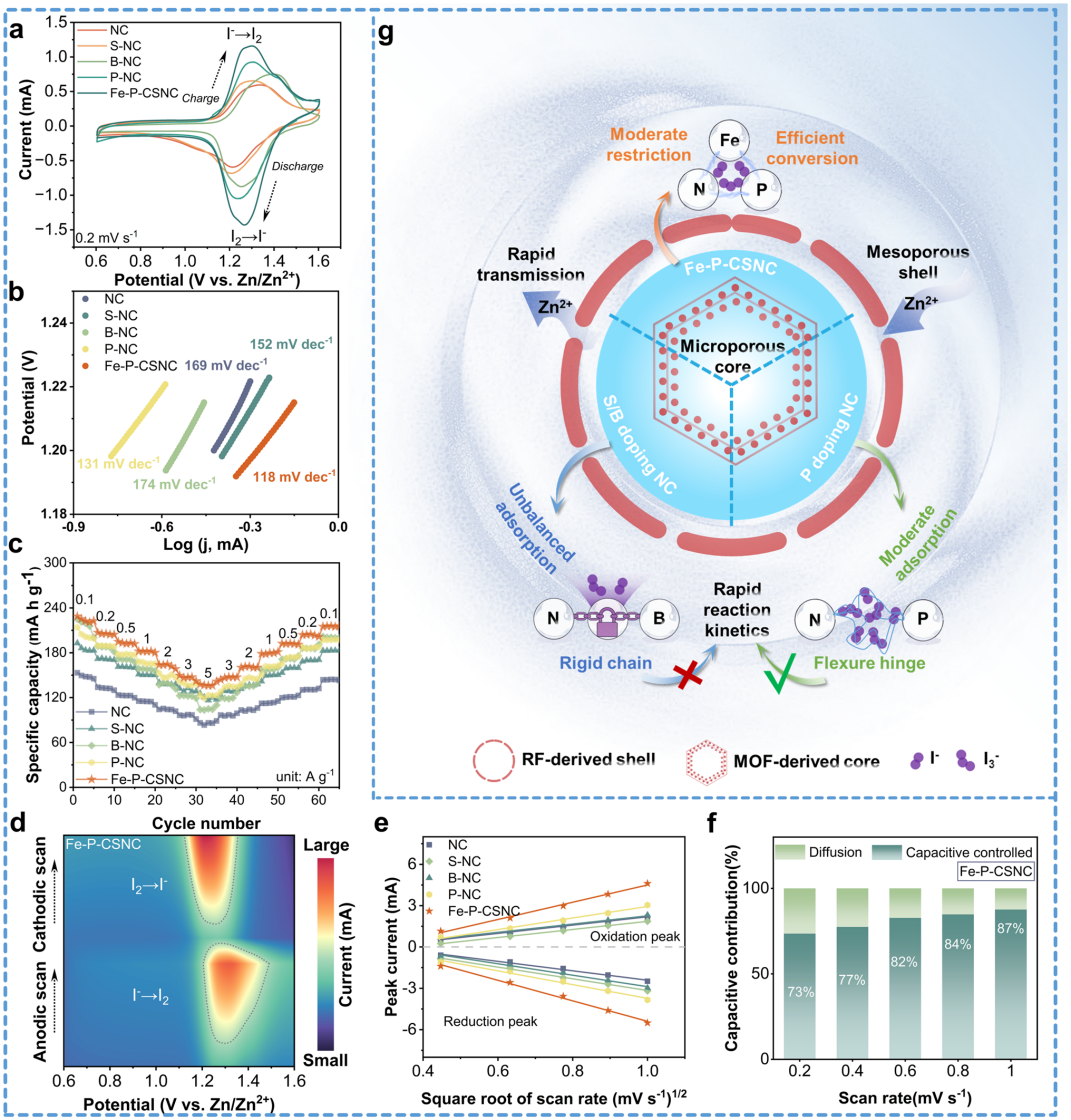
Electrochemical performance of various iodine hosts: **a** CV curves at 0.2 mV s^−1^. **b** Tafel plots of different cathodes during oxidation. **c** rate performance. **d** CV curves of Fe–P-CSNC at different scan rate. **e** linear fits of peak current with the square root of scanning speeds ranging from 0.2 to 1 mV s^−1^. **f** capacitive contribution of Fe–P-CSNC at different scan rate. **g** schematic diagram of the application of heteroatom-doped core–shell structure single-atom catalysts in reaction process

To investigate the impact of heteroatom incorporation on the performance of carbon substrates and single-atom catalysts, we evaluated the electrochemical properties of various samples. The iodine redox behavior of different catalysts was first compared by cyclic voltammetry (CV) tests (Figs. 3a and S7), where all samples show two pairs of redox peaks corresponding to the conversion of I^−^/I_2_ and I_2_/I^−^, NC and S-NC have low peak current and severe polarization, indicating the limited improvement of S doping on catalytic activity. It is worth noting that B doping exhibits certain advantages compared to S-NC during the reduction process, but it is more sluggish during the oxidation process. This may be due to the strong Lewis acid–base coordination between the electron-deficient Lewis acid sites formed by B doping and the electron-rich I^−^ (strong Lewis’s base) during the charging process, which results in the firm anchoring of I^−^ on the active sites. After oxidation, the desorption energy barrier increases, and the products that cannot be quickly desorbed continue to occupy the active sites, leading to site passivation and ultimately causing sluggish oxidation reaction kinetics. P-NC shows significantly better redox reversibility than B/S-doped samples, preliminarily confirming that P is the optimal doping element in the B/S/P system. Notably, the unique electron configuration of P heteroatoms not only enables moderate adsorption of iodine species itself but also provides an ideal electronic modulation platform for the construction of transition metal single-atom active centers via vicinal electronic effect, which cannot be achieved by electro-deficient B and electro-neutral S doping. Fe–P-CSNC exhibits the highest peak current and the most symmetrical peak shape, proving that the strong synergy between P doping and Fe-N_*x*_ sites greatly improves the reaction reversibility. The above CV curves intuitively reflect the differences in redox reversibility of electrodes modified with different heteroatom doping and single atoms. To reveal the oxidation reaction kinetics of the electrodes further quantitatively, we carried out Tafel slope characterization. Tafel plots during oxidation (Fig. 3b) show that the slope of Fe–P-CSNC is only 118 mV dec^−1^, which is much lower than that of M_1_-NC, indicating that the introduction of Fe-N_*x*_ sites enhances the catalytic effect of the material on iodine conversion. B-NC exhibits the slowest reaction kinetics during the oxidation process. The advantage of intrinsic catalytic activity derived from the Fe–P synergy is finally reflected in the rate performance of batteries (Figs. 3c and S8), where Fe–P-CSNC exhibits the highest specific capacity in the full current density range, maintaining a high capacity of 146 mAh g^−1^ at 5 A g^−1^. Compared to NC, the introduction of heteroatoms enhances the capacity release ability under high currents. However, maybe due to the stronger adsorption of B-NC on I^−^, which leads to difficulty in desorption, the battery with B-NC exhibits a more pronounced decline during the increase in current.

To further reveal the kinetic essence of the excellent performance brought by the Fe–P synergy, the reaction reversibility, ion diffusion behavior and capacity contribution source of Fe–P-CSNC were systematically analyzed (Figs. 3d–f, S9, and S10): the CV contour plot in Fig. 3d shows that the two pairs of redox peaks of Fe–P-CSNC are independent and symmetrical without obvious distortion with the increase in scan rate, proving that the iodine redox reaction catalyzed by the Fe–P synergistic active sites has high reversibility and stability; the fitting results of peak current vs. square root of scan rate in Fig. 3f show that the fitting slope of Fe–P-CSNC is much higher than that of other samples (Fig. S11), indicating that its core–shell structure and Fe–P synergistic high active sites synergistically achieve the fastest ion diffusion and interfacial charge transfer [29, 30]; the capacitance contribution analysis in Figs. 3f and S12 shows that the capacitive-controlled capacity of Fe–P-CSNC is dominant at scan rates from 0.2 to 1.0 mV s^−1^, up to 87%, and the high capacitive contribution originates from the highly exposed Fe–P synergistic single-atom sites and hierarchical porous structure, which is the core reason for its excellent rate performance. The corresponding EIS results (Fig. S13) further verify the rapid charge transfer and ion diffusion of Fe–P-CSNC. Figure 3g intuitively reveals the origin of performance differences among different doping systems, where B/S doping cannot achieve precise regulation of adsorption strength, easily leading to unbalanced adsorption and sluggish kinetics, while Fe–P-CSNC realizes rapid transport of Zn^2+^ through its core–shell hierarchical porous structure, and more critically, the synergistic effect between P and Fe centers is achieved as P atoms break the planar symmetry of Fe-N_*x*_ sites through vicinal electronic effect and geometric distortion[14, 31, 32], precisely regulate the electronic structure of the active center, and realize moderate adsorption of polyiodides, which balances the reversible anchoring and efficient catalytic conversion of polyiodides and fundamentally solves the core adsorption-catalysis imbalance problem of B/S doping systems.

**Fig. 4:**
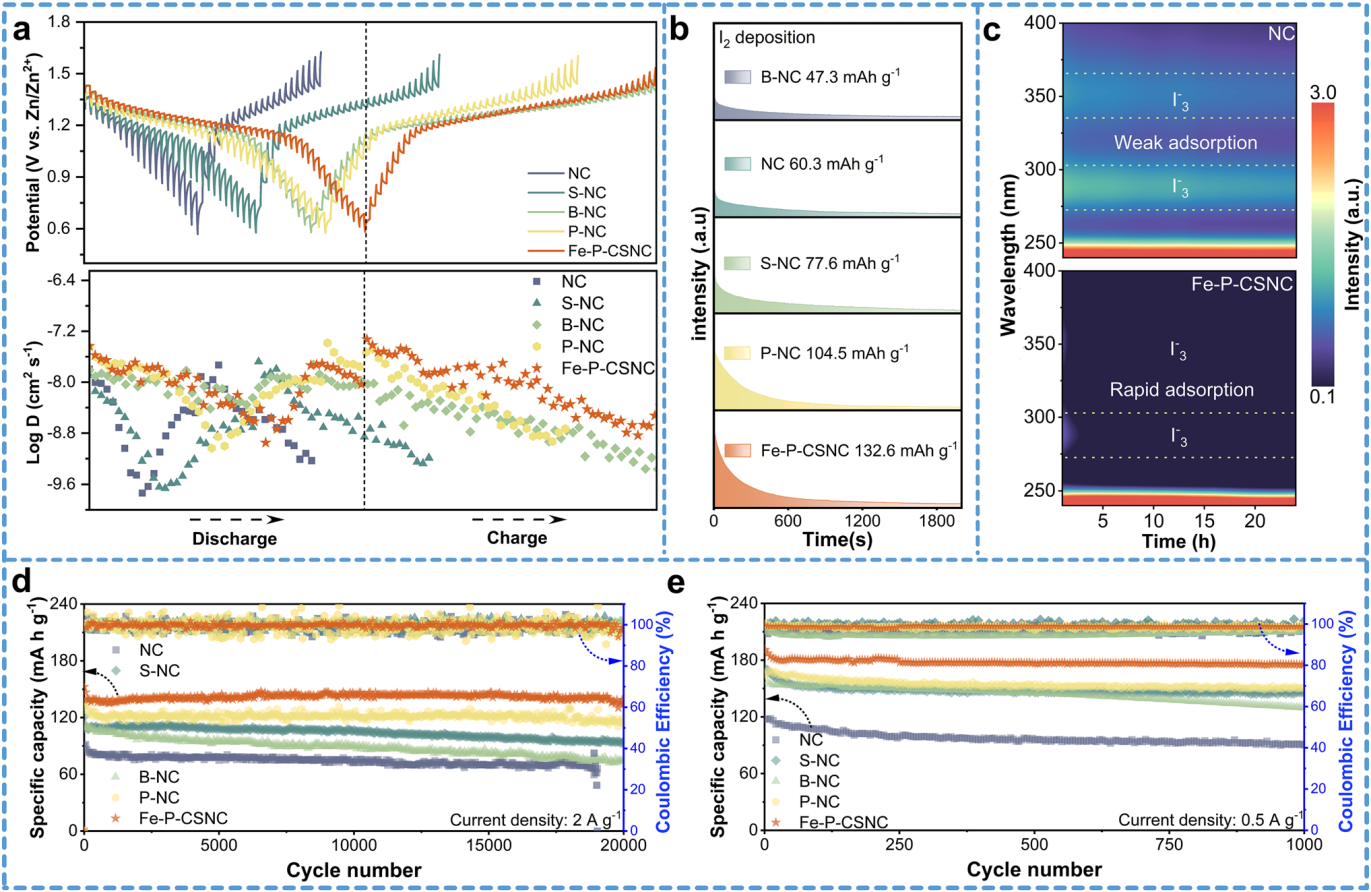
**a** GITT plots and the corresponding diffusion coefficients. **b** Potentiostatic I_2_ deposition curves. **c** In situ monitoring of polyiodide solution adsorption on different materials. Long-term cycling tests of AZIBs with varying catalysts at **d** 2 A g^−1^ and **e** 0.5 A g^−1^

The galvanostatic intermittent titration technique (GITT) was employed to quantitatively evaluate the electrochemical polarization and ionic transport kinetics of the as-prepared electrodes. Throughout the entire charge–discharge process, Fe–P-CSNC exhibits the lowest reaction energy barrier for iodine redox (Fig. 4a), coupled with the highest and least fluctuated Zn^2+^ diffusion coefficient, demonstrating its exceptional kinetic stability [33, 34]. In contrast, the B-NC electrode shows obvious hysteresis during the charging process, which is fully consistent with the severe polarization of the oxidation peak of B-NC in the CV test. To quantify the intrinsic catalytic activity of the cathodes toward the I^−^ oxidation reaction, potentiostatic I_2_ deposition tests were performed on all samples. Fe–P-CSNC delivers the highest I_2_ deposition capacity (Fig. 4b), which corroborates its moderate adsorption strength toward I^−^. Sustained and effective catalysis of the I^−^ oxidation process is made possible by such an adsorption mode, which successfully prevents passivation of active sites. By contrast, the electron-rich I^−^ forms strong, persistent connections with the electron-deficient B sites, resulting in the sustained occupancy of active sites and the suppression of I^−^ oxidation [35].

In situ UV–Vis absorption spectroscopy was further conducted to directly visualize the concentration variation of free I_3_^−^ in the electrolyte during the resting process (Figs. 4c andS14). The characteristic absorption peak of I_3_^−^ (280 nm) in the electrolyte with Fe–P-CSNC decays rapidly within a short time, exhibiting a distinct rapid adsorption behavior. This confirms that the electron-rich Lewis base sites constructed by P doping possess strong adsorption capacity toward I_3_^−^, which can rapidly confine the dissolved polyiodides generated during charge–discharge to the cathode side, thus fundamentally inhibiting the notorious shuttle effect. In contrast, the electrolyte with NC powder still maintains a high intensity of the I_3_^−^ characteristic absorption peak after long-time resting, indicating its weak adsorption capacity toward polyiodides. After charging the batteries to 1.6 V and standing for 24 h, the capacity retention rate of the Fe–P-CSNC/I_2_ battery is 91.3%, while that of the NC/I_2_ battery is only 83.6% (Fig. S15). This result further proves that Fe–P-CSNC has excellent anchoring ability to polyiodides and can effectively inhibit self-discharge.

Long-cycle performance is a core metric for evaluating the practical application potential of the catalysts in AZIBs, where capacity retention and Coulombic efficiency directly reflect the structural stability and anti-poisoning ability of the active sites. At a high current density of 2 A g^−1^ (Fig. 4d), Fe–P-CSNC delivers the highest discharge specific capacity, with no obvious capacity decay after an ultra-long 20,000 cycles, and its Coulombic efficiency remains close to 100% throughout cycling. In contrast, B-NC exhibits the fastest capacity decay due to its intrinsically sluggish oxidation kinetics.

It is worth noting that the single-cycle charge–discharge duration is significantly prolonged at low current density, which greatly improves the conversion depth and completeness of iodine species. Under this condition, I_3_^−^ with higher concentration are generated at the cathode side and exist for a longer time, providing a sufficient concentration gradient and time window for the cross-membrane diffusion of polyiodides toward the Zn anode, thus imposing a more stringent test on cathode performance. After 1000 cycles at 0.5 A g^−1^ (Fig. 4e), Fe–P-CSNC exhibits the lowest capacity decay rate of only 0.05% per cycle, which is highly consistent with its excellent polyiodide adsorption performance revealed by the in-situ UV–vis test (Fig. 4c). In addition, we performed SEM characterization of the cycled Zn anodes, and the results show that the surface of the Zn anode in the battery using the Fe–P-CSNC cathode is relatively smoother (Fig. S16).

Although B-NC delivers a higher initial capacity than S-NC and NC, the strong adsorption of I^−^ by B sites induces continuous site poisoning during the prolonged charging process at low current density. The active sites are progressively passivated and deactivated during cycling, which eventually leads to severe capacity decay, verifying the adverse effect of unbalanced adsorption predicted by our theoretical analysis. By contrast, Fe–P-CSNC achieves excellent cycling stability through the moderate adsorption design, exhibiting great practical application potential. The AZIBs’ performance is on par with and often even better than that of previously reported iodine cathodes (Table S2). The assembled pouch cells based on Fe–P-CSNC show no obvious capacity degradation after 100 cycles, and three tandem cells can continuously power a commercial LED array, demonstrating its feasibility in practical portable energy storage scenarios (Fig. S17).

The electrochemical results demonstrate the notable performance difference between various heteroatom-doped and single-atom-decorated samples and provide evidence that P doping and Fe single-atom incorporation can successfully improve the iodine cathode’s redox kinetics and cycling stability. We also conducted charge density difference calculations, partial density of states (PDOS) analysis in conjunction with *d*-band center theory, and systematically examined the electronic structure modulation of various heteroatoms and metal centers in order to uncover the intrinsic electronic origin of the observed performance difference and validate the central hypothesis of moderate adsorption design (Figs. S18 and S19). Meanwhile, in situ characterizations were performed to directly view the dynamic development of polyiodides and interfacial reaction kinetics during charge–discharge cycling.

**Fig. 5:**
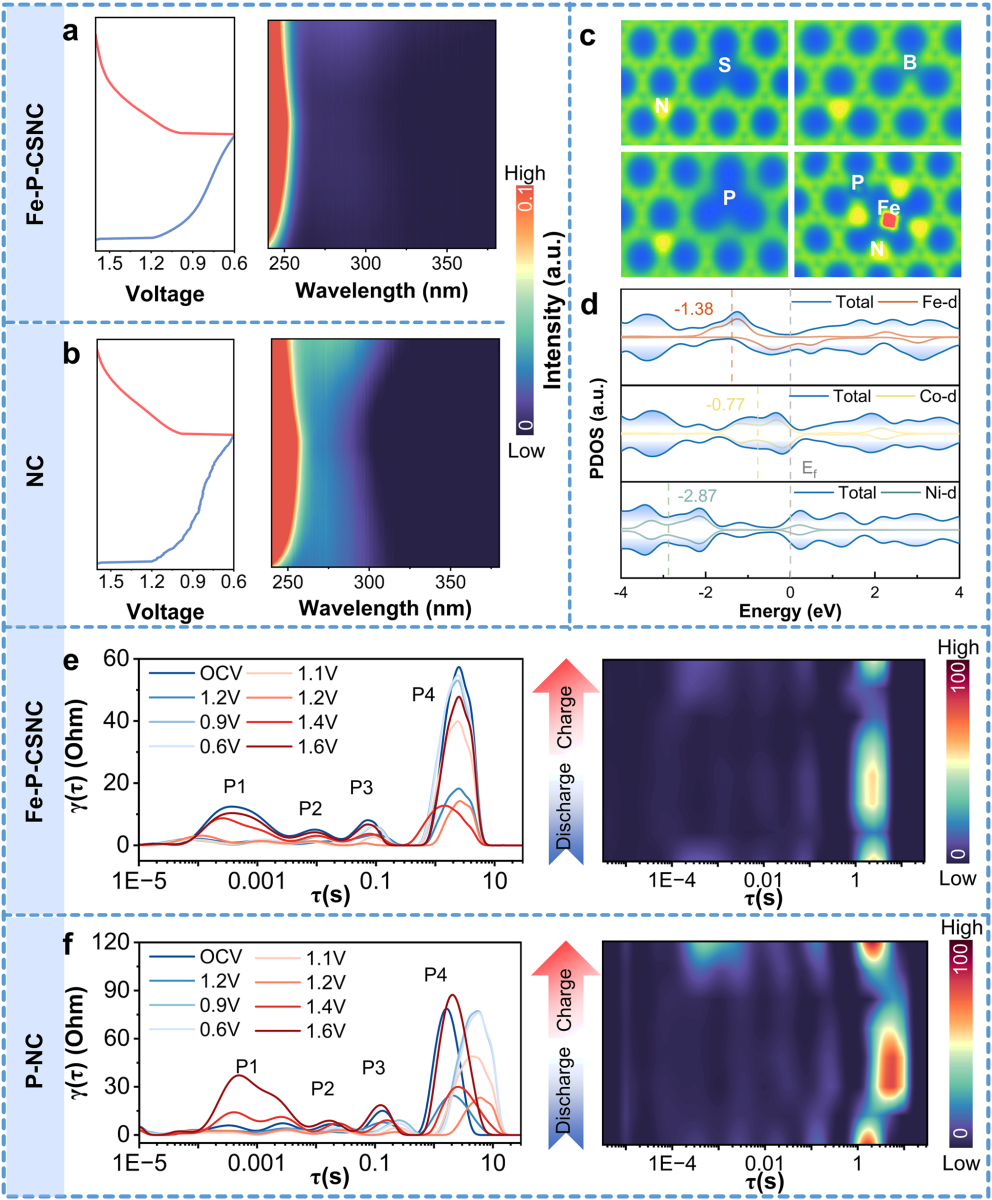
**a-b** In situ UV–vis spectra of batteries during cycling process. **c** differential charge density of different samples. **d** PDOS of M_2_-P-CSNC. **e–f** DRT calculated from EIS measurements of various batteries at different voltages and corresponding contour plots

The dynamic evolution of soluble polyiodide intermediates during charge–discharge cycling was visualized in real time via in situ UV–vis absorption spectroscopy (Fig. 5a, b). Due to the lack of effective suppression of the shuttle effect in NC, the concentration of I_3_^−^ in the electrolyte experiences significant fluctuations as charging and discharging progress [36]. By comparison, Fe–P-CSNC achieves further optimized performance through the triple synergistic effect of polar anchoring from P doping, chemical adsorption, and catalytic activation from atomically dispersed Fe sites. Only negligible reversible fluctuation of the I_3_^−^ absorption peak is observed throughout the full charge–discharge process without obvious intensity elevation.

The performance disparity between B/S-NC and P-NC is intrinsically rooted in the electronic structure modulation of the carbon matrix, as revealed by charge density difference calculations (Figs. 5c and S20). Distinct electron depletion regions emerge around the doped P atoms, with the corresponding charge perturbation extending across multiple carbon rings adjacent to the P sites. This observation demonstrates that P doping can effectively disrupt the conjugated electroneutrality of the carbon matrix and induce pronounced localized charge redistribution, thereby providing an optimal electronic structural foundation for the construction of single-atom active centers [14]. The synergistic incorporation of P doping and Fe single atoms enables the successful construction of a strongly localized, electron-enriched active center centered on the Fe site in Fe–P-CSNC. This tailored active center not only realizes reversible anchoring of polyiodides via a localized polarized electric field to mitigate the shuttle effect, but also furnishes highly active catalytic sites for the bidirectional I^−^/I_2_ redox reaction.

Beyond electronic structure modulation, the adsorption behavior of iodine species on transition metal single-atom sites was investigated via PDOS calculations combined with d-band center theory (Fig. 5d). The calculation results reveal that the d-band center of the Fe site is located in the optimal moderate adsorption interval, which is well consistent with the electrochemical test results (Figs. S7–S11).

The theoretically predicted superior catalytic activity and reaction kinetics of Fe–P-CSNC were further validated at the electrochemistry level using in-situ EIS coupled with the distribution of relaxation times (DRT) method. We deconvoluted the impedance evolution of the electrode over the potential window of 0.6–1.6 V, and four characteristic relaxation peaks (P1 to P4) were obtained (Fig. 5e, f). The P1 peak corresponds to ionic transport within the interfacial layer at the electrode/electrolyte interface. The merged P2-P3 region is assigned to the charge-transfer process of the bidirectional I^−^/I_2_ redox. The P4 peak is attributed to mass transport of the electrolyte phase in electrolyte-filled pores. The corresponding contour maps directly visualize the impedance evolution of different relaxation processes throughout the whole cycling process [33]. The peak intensity of P1 directly reflects the ionic transport resistance within the electrode/electrolyte interfacial layer. The NC electrode exhibits the highest P1 peak intensity with obvious fluctuation along with potential variation (Fig. S21), indicating a large ionic transport resistance within the interfacial layer of the unmodified carbon host [37]. The optimized surface polarity induced by P doping improves the interfacial compatibility between the electrode and electrolyte, thus moderately reducing the interfacial ionic transport resistance of the P-NC electrode. In contrast, the Fe–P-CSNC electrode maintains the lowest P1 peak intensity throughout the full potential window, as the synergistic effect between Fe single atoms and P doping further optimizes the electrode/electrolyte interfacial structure and enables rapid ionic transport [5].

As the rate-determining step of the overall electrochemical reaction, the impedance variation in the P2-P3 region directly reflects the intrinsic catalytic activity and redox kinetics of the electrode. For the NC electrode, the peak intensity in the P2-P3 region rises sharply in both the 1.2–0.9 V (dominated by I_2_ reduction) and 1.1–1.4 V (dominated by I^−^/I_2_ oxidation) [38]. This demonstrates that the pure carbon host has no intrinsic catalytic activity and cannot effectively reduce the charge-transfer energy barrier of the I^−^/I_2_ redox reaction. The P-NC electrode shows a significantly decreased peak intensity in the P2-P3 region compared with NC, as the surface polarity regulation induced by P doping optimizes the adsorption behavior of iodine species on the carbon host and moderately reduces the charge-transfer resistance. However, an elevation of peak intensity is still observed in the core charge–discharge plateau window. This indicates that the polar effect of P doping alone cannot offer dedicated catalytic active sites to fully overcome the reaction energy barrier, making it difficult to fundamentally eliminate the kinetic limitation, and the bidirectional reaction kinetics still exhibit moderate sluggishness and imbalance. Conversely, no obvious impedance jump is observed in the P2-P3 region of the Fe–P-CSNC electrode throughout the full potential window. This result directly confirms that the electronic synergistic effect between Fe single atoms and P doping precisely regulates the electronic structure of the Fe-N_*x*_ active center. Through the moderate adsorption effect predicted by d-band center theory, it not only provides sufficient electrons for I-I bond activation and significantly reduces the charge-transfer energy barrier of the bidirectional redox, but also achieves a well-balanced bidirectional kinetic between the reduction and oxidation processes. Meanwhile, the spatial isolation effect of the core–shell cavity structure further avoids direct contact between the highly active Fe-N_*x*_ sites and electrolyte byproducts, thus fundamentally preventing irreversible poisoning of the active sites and guaranteeing long-term stable catalytic activity from the structural level.

For the P4 peak corresponding to polyiodide mass transport and solid iodine phase conversion in the pores, its impedance variation is directly correlated with the suppression efficiency of the polyiodide shuttle effect and the long-term cycling stability of the electrode. The NC electrode shows a sharp surge of P4 peak intensity in the deep discharge and charge regions, indicating that the pore structure of the pure carbon host has no effective confinement and anchoring effect on soluble polyiodides. The extremely large mass transport resistance of polyiodides in the pores easily triggers a severe shuttle effect, and the irreversible I_2_ deposition/dissolution process tends to cause a vicious cycle of pore blockage and mass transport retardation [35]. The polar anchoring effect induced by P doping can restrict the free diffusion of polyiodides to a certain extent and alleviate the shuttle effect, but a significant impedance elevation still exists in the deep charge–discharge regions. This demonstrates that the polar effect of P doping alone cannot achieve long-term stable confinement of polyiodides and highly efficient I_2_ conversion, and there remains a risk of mass transport retardation and aggravated shuttle effect during long-term cycling. By comparison, the hierarchical pore-cavity structure of the Fe–P-CSNC core–shell electrode enables simultaneous fast mass transport and spatial confinement of polyiodides, which, combined with the polar anchoring effect of P doping, fundamentally inhibits the polyiodide shuttle effect. The high catalytic activity of Fe single-atom sites further ensures the high reversibility of the I_2_ deposition/dissolution process, effectively avoiding pore blockage and mass transport retardation, and providing direct kinetic support for the ultra-long cycling stability of the electrode.

## Conclusions

In conclusion, through systematic screening of ZIF-8-derived carbon hosts with single-heteroatom doping (B, S, and P), this work identifies P doping as the optimal strategy to balance the adsorption of polyiodide intermediates and desorption of iodine species, enabling stable and reversible redox cycling in AZIBs.

Building on this finding, we develop a facile and universal phytic acid-assisted in situ synthetic strategy to fabricate core–shell structured single-atom catalysts (M_2_-P-CSNC) via a one-pot process. Leveraging the dual role of phytic acid as both the phosphorus source and in situ etchant, this strategy synchronously realizes in situ P doping, the construction of hierarchical core–shell architecture, and atomic dispersion of transition metal centers. The unique core–shell structure serves as a robust nanoreactor for iodine species, integrating sufficient active site exposure, stable polyiodide confinement, and rapid ion/electron transport. Meanwhile, the in situ incorporated P heteroatoms precisely tailor the local electronic environment and d-band center position of the M-N_*x*_ active centers, further optimizing the adsorption strength of iodine intermediates to the optimal moderate range, thus achieving well-balanced bidirectional redox catalysis.

Benefiting from the synergistic structural and electronic effects, the optimized Fe–P-CSNC electrode delivers outstanding rate capability over a wide current density range, along with exceptional long-term cycling stability with negligible capacity decay after 20,000 cycles at 2 A g^−1^. The moderate adsorption design principle and the facile in situ heteroatom doping strategy for local electronic modulation of single-atom active centers proposed in this work provide a rational guideline for addressing the intermediate shuttle effect and unbalanced bidirectional redox kinetics in various conversion-type energy storage systems.

## Supplementary Information

Below is the link to the electronic supplementary material.

Supplementary file1 (DOCX 5866 KB)

## References

[1] X. Yang, H. Fan, F. Hu, S. Chen, K. Yan et al., Aqueous zinc batteries with ultra-fast redox kinetics and high iodine utilization enabled by iron single atom catalysts. Nano-Micro Lett. **15**(1), 126 (2023). 10.1007/s40820-023-01093-710.1007/s40820-023-01093-7PMC1019999837209237

[2] Y. Wang, X. Fan, E. Liu, H. Li, Q. Wei, Constructing carbon-based perovskite solar cells based on the energy bands of perovskite synergistically optimized by graphite fluorinated polymers. Chem. Eng. J. **542**, 177954 (2026). 10.1016/j.cej.2026.177954

[3] Y. Fan, W. Qu, K. Xu, X. Wang, J. Dai et al., Multi-polar order engineering enables near-ideal efficiency in lead-free energy storage perovskite. Adv. Mater. **38**, e18270 (2026). 10.1002/adma.20251827041392393 10.1002/adma.202518270

[4] R.-Q. Liu, W.-S. Shang, J.-T. Zhang, Bridging materials and energy storage mechanisms in Zn-I_2_ batteries. J. Electrochem. **31**(9), 2515005 (2025). 10.61558/2993-074x.3567

[5] D.-Q. Cai, H. Xu, T. Xue, J.-L. Yang, H.-J. Fan, A synchronous strategy to Zn-iodine battery by polycationic long-chain molecules. Nano-Micro Lett. **18**(1), 3 (2025). 10.1007/s40820-025-01854-610.1007/s40820-025-01854-6PMC1227104540676288

[6] M. Du, P. Geng, C. Pei, X. Jiang, Y. Shan et al., High-entropy Prussian blue analogues and their oxide family as sulfur hosts for lithium-sulfur batteries. Angew. Chem. Int. Ed. **61**(41), e202209350 (2022). 10.1002/anie.20220935010.1002/anie.20220935036006780

[7] C. Liu, Y. Bai, W. Li, F. Yang, G. Zhang et al., In situ growth of three-dimensional MXene/metal–organic framework composites for high-performance supercapacitors. Angew. Chem. Int. Ed. **61**(11), e202116282 (2022). 10.1002/anie.20211628210.1002/anie.20211628235005827

[8] Z. Wu, W. Wang, Y. Wang, C. Chen, K. Li et al., Three-dimensional graphene hollow spheres with high sulfur loading for high-performance lithium-sulfur batteries. Electrochim. Acta **224**, 527–533 (2017). 10.1016/j.electacta.2016.12.072

[9] L. Ni, J. Gu, X. Jiang, H. Xu, Z. Wu et al., Polyoxometalate-cyclodextrin-based cluster-organic supramolecular framework for polysulfide conversion and guest–host recognition in lithium-sulfur batteries. Angew. Chem. Int. Ed. **62**(36), e202306528 (2023). 10.1002/anie.20230652810.1002/anie.20230652837464580

[10] S. Li, Y. Nie, Y. Wang, G. Feng, Q. Li et al., Quantum size effect synergizes space-limited domain action for advanced aqueous zinc-iodine batteries. Adv. Mater. **38**(4), e14577 (2026). 10.1002/adma.20251457741185985 10.1002/adma.202514577PMC12810656

[11] Y. Luo, M. Wu, D. Zhang, J. Liu, Y. He et al., Boosted polysulfide conversion by Co, Mn bimetallic-modulated nitrogen–carbon material for advanced lithium–sulfur batteries. ACS Sustain. Chem. Eng. **11**(3), 1087–1099 (2023). 10.1021/acssuschemeng.2c06029

[12] L. Ni, G. Zhao, Y. Wang, Z. Wu, W. Wang et al., Coaxial carbon/MnO_2_ hollow nanofibers as sulfur hosts for high-performance lithium-sulfur batteries. Chem. Asian J. **12**(24), 3128–3134 (2017). 10.1002/asia.20170134329045068 10.1002/asia.201701343

[13] J. Li, G. Ni, Z. Cheng, J. He, F. Cao et al., Synergistic structural, chemical, and catalytic modulation of carbon host for high-performance aqueous zinc–iodine batteries. Electrochim. Acta **540**, 147258 (2025). 10.1016/j.electacta.2025.147258

[14] S. Huang, Z. Hu, X. He, L. Cao, M. Ye et al., Phosphorus-induced charge redistribution and lattice self-regulation in Cu_3_PSe_4_ enables low N/P ratio and durable Zn–I_2_ batteries. Angew. Chem. Int. Ed. **65**(10), e23544 (2026). 10.1002/anie.20252354410.1002/anie.20252354441612713

[15] C. Dong, Y. Yu, C. Ma, C. Zhou, J. Wang et al., Tailoring zinc diatomic bidirectional catalysts achieving orbital coupling–hybridization for ultralong-cycling zinc–iodine batteries. Energy Environ. Sci. **18**(6), 3014–3025 (2025). 10.1039/d4ee05767h

[16] X. Guo, H. Xu, Z. Qiu, Q. Li, N. Li et al., Heteroatom-modulated asymmetric cobalt single-atom catalysts on MOF-derived carbon enabling durable zinc-iodine batteries. Adv. Mater. **37**(45), e14035 (2025). 10.1002/adma.20251403540847723 10.1002/adma.202514035

[17] Z. Chen, J. Yang, R. Li, B. Yan, P. Chen et al., Boosting iodine redox kinetics by nickel-cobalt diatomic electrocatalyst for zinc-iodine batteries. Small **21**(23), 2500936 (2025). 10.1002/smll.20250093610.1002/smll.20250093640270340

[18] L. Zhu, X. Guan, Z. Zhang, Y. Fu, Z. Yuan et al., Catalytic materials for energy applications. Chem. Commun. **61**, 961 (2025). 10.1039/D4CC06281G

[19] Z. Lu, J. Zheng, P. Chen, Y. Yang, J. He et al., Electron localization-stabilized inherent multivalence states enable D-band center upshift promoting high redox activity for sodium storage in Cu-based VI-group sulfides. Adv. Funct. Mater. **36**(41), e28754 (2026). 10.1002/adfm.202528754

[20] B. Sun, D. Wang, Y. Jiang, R. Wang, L. Lyu et al., Cyclodextrin metal–organic framework functionalized carbon materials with optimized interface electronics and selective supramolecular channels for high-performance lithium–sulfur batteries. Adv. Mater. **36**(52), 2415633 (2024). 10.1002/adma.20241563310.1002/adma.20241563339501988

[21] S. Zhang, X. Zhou, G. Zhou, B. He, H. Pang et al., Template-assisted fabrication of O-doped CoP microflowers with optimal electronic modulation for electrochemical hydrogen evolution. Chem-Eur. J. **29**(41), e202301252 (2023). 10.1002/chem.20230125237194695 10.1002/chem.202301252

[22] J. Wang, X. Guo, Q. Jing, W. Li, T. Chen et al., Rational design of self-sacrificial template derived quasi-Cu-MOF composite as anodes for high-performance lithium-ion batteries. Chin. Chem. Lett. **34**(6), 107675 (2023). 10.1016/j.cclet.2022.07.018

[23] P. Geng, M. Du, C. Wu, T. Luo, Y. Zhang et al., PPy-constructed core–shell structures from MOFs for confining lithium polysulfides. Inorg. Chem. Front. **9**(10), 2389–2394 (2022). 10.1039/d2qi00392a

[24] Y. Jiang, M. Du, P. Geng, B. Sun, R. Zhu et al., CoO/MoO_3_@Nitrogen-doped carbon hollow heterostructures for efficient polysulfide immobilization and enhanced ion transport in Lithium-Sulfur batteries. J. Colloid Interface Sci. **664**, 617–625 (2024). 10.1016/j.jcis.2024.03.01538490037 10.1016/j.jcis.2024.03.015

[25] Y. Liu, J. Han, L. Fan, Y. Li, R. Guo, Pomegranate-like multicore-shell Mn_3_O_4_ encapsulated mesoporous N-doped carbon nanospheres with an internal void space for high-performance lithium-ion batteries. Chem. Commun. **55**(56), 8064–8067 (2019). 10.1039/c9cc03727f10.1039/c9cc03727f31237580

[26] S. Zheng, X. Guo, H. Xue, K. Pan, C. Liu et al., Facile one-pot generation of metal oxide/hydroxide@metal–organic framework composites: highly efficient bifunctional electrocatalysts for overall water splitting. Chem. Commun. **55**(73), 10904–10907 (2019). 10.1039/c9cc06113d10.1039/c9cc06113d31441472

[27] Y. Wu, N. Wu, X. Jiang, S. Duan, T. Li et al., Bifunctional K_3_PW_12_O_40_/graphene oxide-modified separator for inhibiting polysulfide diffusion and stabilizing lithium anode. Inorg. Chem. **62**(38), 15440–15449 (2023). 10.1021/acs.inorgchem.3c0172037700509 10.1021/acs.inorgchem.3c01720

[28] S.-J. Zhang, J. Hao, H. Wu, Q. Chen, Y. Hu et al., Coordination chemistry toward advanced Zn-I_2_ batteries with four-electron I^−^/I^0^/I^+^ conversion. J. Am. Chem. Soc. **147**(19), 16350–16361 (2025). 10.1021/jacs.5c0208540325936 10.1021/jacs.5c02085

[29] D. Wang, X. Wan, J. Wang, D. Mangelings, Q. Xu et al., Applicability of core-shell SiO_2_ microspheres with a high TiO_2_ loading as stationary phase for HPLC. Anal. Chim. Acta **1272**, 341527 (2023). 10.1016/j.aca.2023.34152737355322 10.1016/j.aca.2023.341527

[30] M. Ye, F. Shi, M. Shen, W. Qin, C. Ren et al., Composite soft-template method synthesis and biosensing application of hedgehog-like bismuth sulfide micro-nanostructures. Colloids Surf. A Physicochem. Eng. Asp. **613**, 126094 (2021). 10.1016/j.colsurfa.2020.126094

[31] B. Xie, X. Wu, J. Wang, R. Wang, Y. Dong et al., Confinement sacrifice template synthesis of size controllable heterogeneous double-layer hollow spheres SnO_2_@Void@HCSs as anode for Li^+^/Na^+^ batteries. J. Electroanal. Chem. **923**, 116830 (2022). 10.1016/j.jelechem.2022.116830

[32] J. Wang, J. Han, C. Zhu, N. Han, J. Xi et al., Gold nanorods/polypyrrole/m-SiO_2_ core/shell hybrids as drug nanocarriers for efficient chemo-photothermal therapy. Langmuir **34**(48), 14661–14669 (2018). 10.1021/acs.langmuir.8b0266730398351 10.1021/acs.langmuir.8b02667

[33] Y. Qi, Q. Liu, B. Wang, C. Wang, Z. Chen et al., Role of high-defect biocarbon for achieving iodine efficient anchoring and rapid conversion in zinc-iodine battery. Phys. Scr. **100**(8), 085995 (2025). 10.1088/1402-4896/adfabe

[34] C. Li, H. Li, X. Ren, L. Hu, J. Deng et al., Urea chelation of I^+^ for high-voltage aqueous zinc–iodine batteries. ACS Nano **19**(2), 2633–2640 (2025). 10.1021/acsnano.4c1445139772449 10.1021/acsnano.4c14451

[35] T. Xiao, J.-L. Yang, R.-J. Xu, H. Xu, H. Liu et al., Balanced iodophilicity and solvophilicity unlocks fast iodine conversion chemistry. J. Am. Chem. Soc. **147**(32), 28820–28830 (2025). 10.1021/jacs.5c0578640750094 10.1021/jacs.5c05786PMC12356581

[36] X. Huang, S. Zhao, S. Yang, X. Wang, L. Liu et al., Dynamic interhalogen coupling engineered by multifunctional ionic liquid for high-energy aqueous Zn-I2 batteries. Adv. Funct. Mater. **36**(14), e19437 (2026). 10.1002/adfm.202519437

[37] X. Liang, Q. Dong, S. Guo, C. Zeng, Z. Chen et al., Customized design of R-SO_3_H-containing binders for durable iodine-loading cathode of zinc–iodine batteries. Adv. Energy Mater. **15**(28), 2500673 (2025). 10.1002/aenm.202500673

[38] Z. Chen, X. Gao, L. Shan, Q. Fu, Z. Xing et al., Taming polyiodides: phenol chemistry for shuttle-free and durable zinc–iodine batteries. Energy Environ. Sci. **18**(19), 8768–8779 (2025). 10.1039/d5ee02763b

