# Supplementary material for: From Screening to Site Control: Phytic-Acid Mediated P-Tuning of M–N Coordination to Balance Iodine Adsorption and Stability in Zn–I2 Batteries
Source: Nanomicro Lett. 2026 Jul 30;19:10. doi: 10.1007/s40820-026-02321-6 (PMC13424055; doi:10.1007/s40820-026-02321-6)
Supplement: Supplementary file 1 — Supplementary file1 (DOCX 5866 KB) [file 40820_2026_2321_MOESM1_ESM.docx]

Supporting Information for

**From Screening to Site Control: Phytic-Acid Mediated P-Tuning of M-N Coordination to Balance Iodine Adsorption and Stability in Zn-I_2_ Batteries**

Yuxuan Jiang^1^, Bingxin Sun^1^, Mohsen Shakouri^2^, Bin He^3^, Wang Zhang^1^, Ran Wang^1,4^, Tianxiao Sun^5^ and Huan Pang^1,^ *

^1^School of Chemistry and Materials, Yangzhou University, Yangzhou 225002, P. R. China

^2^Canadian Light Source, University of Saskatchewan, Saskatoon, Saskatchewan, S7N 2V3, Canada

^3^Zhejiang Key Laboratory for Industrial Solid Waste Thermal Hydrolysis Technology and Intelligent Equipment, Department of Materials Engineering, Huzhou Normal University, Huzhou 313000, P. R. China

^4^Yangzhou Key Laboratory of Smart Materials and Clean Energy, Interdisciplinary Research Center for Advanced Energy, Yangzhou University, Yangzhou 225002, P. R. China

^5^Shanghai Synchrotron Radiation Facility, Shanghai Advanced, Research Institute, Chinese Academy of Sciences, Shanghai 201204, P. R. China

* Corresponding author. E-mail: [huanpangchem@hotmail.com](mailto:huanpangchem@hotmail.com) (Huan Pang)

**S1 Supplementary Text**

***S1.1 Materials***

The Powder X-ray diffraction (PXRD) patterns were performed by Bruker AXS D8 advance with Cu Kα radiation of 40 kV (λ=1.5418 Å). Scanning electron microscopy (SEM) images were obtained by Zeiss-Supra 55 microscope. Transmission electron microscopy (TEM) was recorded using Tecnai G2 F30 S-TWIN at an acceleration voltage of 300 kV. Raman spectroscopy was obtained by using Renishaw InVia Reflex (514 nm laser). Nitrogen sorption isotherms were carried out using a BELSORP-mini (BEL, Japan). The specific surface area (SSA) was analyzed by Multipoint Brunauer-Emmett-Teller (BET) technique. X-ray photoelectron spectroscopy (XPS) analysis was carried out using a Thermo Scientific ESCALAB 250Xi X-ray photoelectron spectrometer with Al Kα radiation of 1486.6 eV as the excitation source. The survey thickness is 2-3 nm. The concentration variations in these solutions were detected by the UV-vis spectroscopy. Synchrotron radiation-based soft X-ray absorption near-edge structure (XANES) characterizations were conducted at BL02B in the Shanghai Synchrotron Radiation Facility (SSRF).

***S1.2 Battery assembling and test***

The CR 2032-type coin cells were fabricated using the working electrode, zinc foil as anode electrode, glass fiber as the separator. The electrolyte is composed of 2M ZnSO_4_ and (100 μL per cell). The GCD tests were estimated in the voltage window of 0.6-1.6 V. The rate capability was also tested by varying the current density from 0.1 A/g to 5 A/g on a battery measurement system (CT2001A, Wuhan Land, China) at room temperature. CV and EIS curves were measured on an electrochemical workstation (CHI660E, Chenhua, Shanghai, China). CV curves was performed from 1.6 V to 0.6 V at a scanning rate of 0.2 mV s^-1^, and the frequency of EIS was performed form 100 kHz to 0.01 Hz at open-circuit potential. For In situ EIS measurements, Zn-iodine cells were discharged in galvanostatic mode at 0.1 A/g for 10 min, and then the cell was rest for 10 min to reach the quasi-open circuit voltage (QOCV). The EIS test is performed when the QOCV is slightly below the target voltage.

***S1.3 Computation methods***

All calculations were implemented with the CASTEP code. The Perdew-Burke-Ernzerhof (PBE) functional of the generalized gradient approximation (GGA) was used to calculate the exchange-correlation energy. The ultrasoft pseudopotential for every atom was adopted. The cutoff energy was 500 eV, the Γ-centered k-mesh was adopted as 3×3×1 for the geometry optimization of all structures. The convergence tolerances of energy change, maximum force, and maximum displacement were set as 2×10^-6^ eV/atom, 0.05 eV/Å, and 0.002 Å, respectively. To eliminate interactions between adjacent unit cells, a vacuum separation >15 Å was employed.

**S2 Supporting Figures**


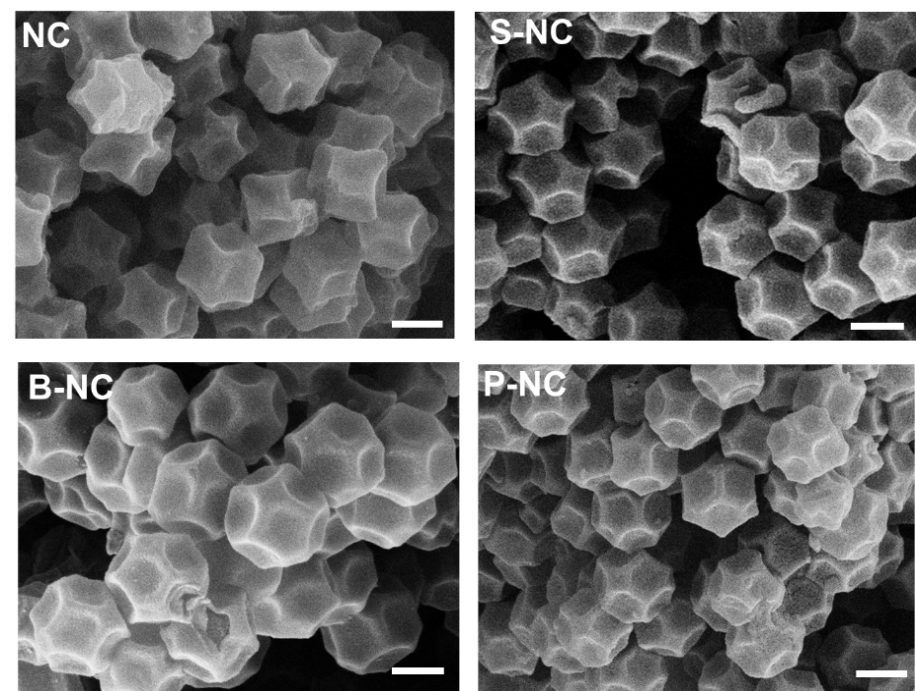


**Fig. S1** SEM images of M_1_-NC (the scale bars in the graphs are 400 nm).


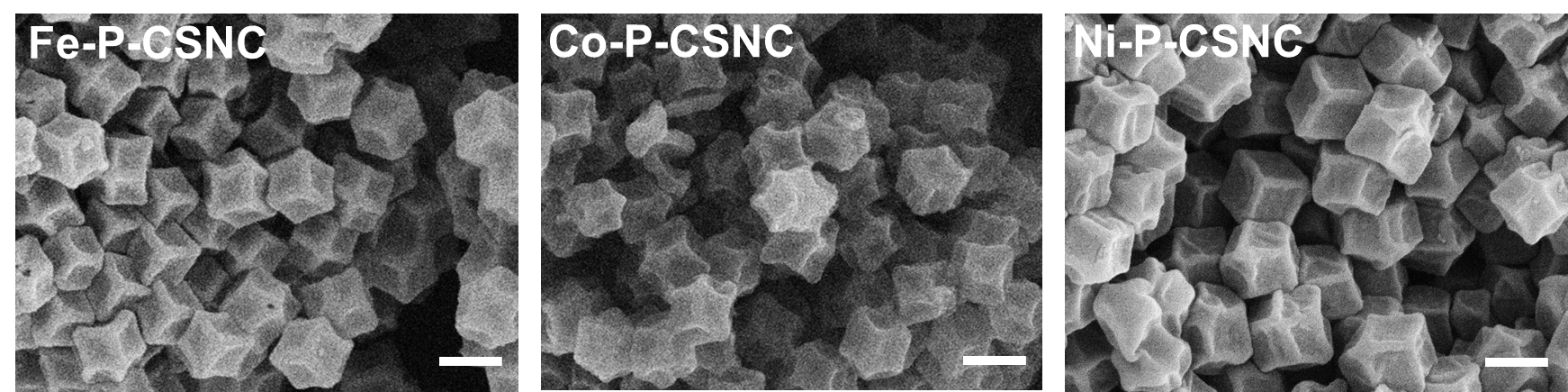


**Fig. S2** SEM images of M_2_-P-CSNC (the scale bars in the graphs are 400 nm).


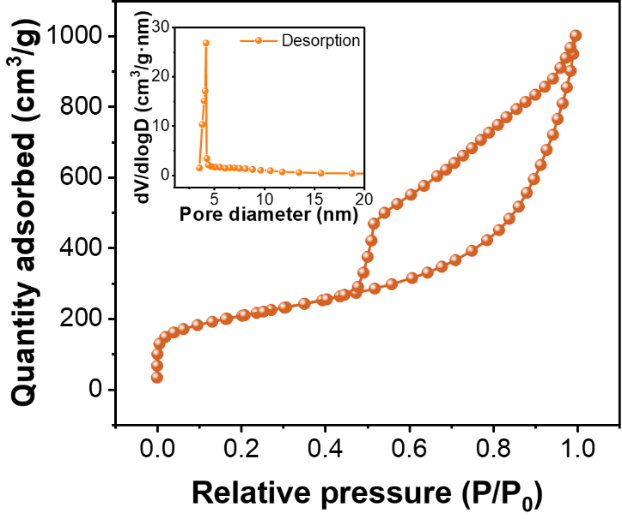


**Fig. S3** Nitrogen adsorption isotherms and pore size distribution of Fe-P-CSNC.


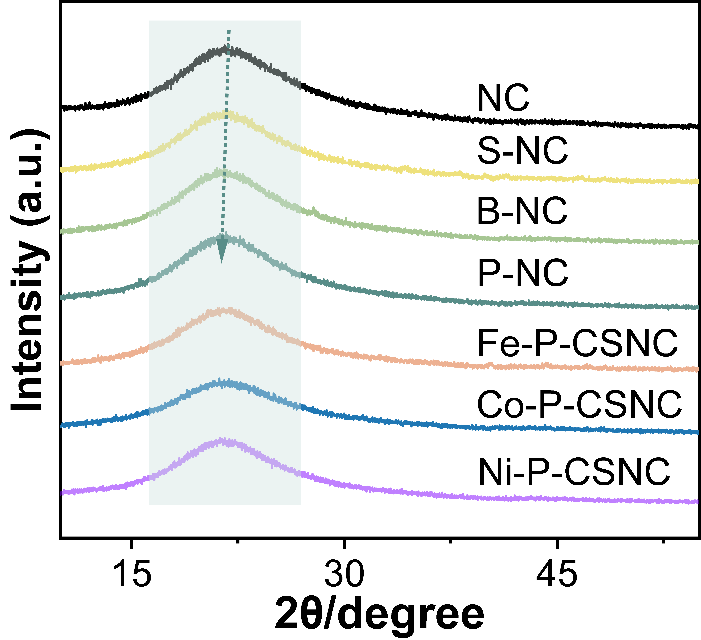


## Fig. S4 XRD patterns of different samples.


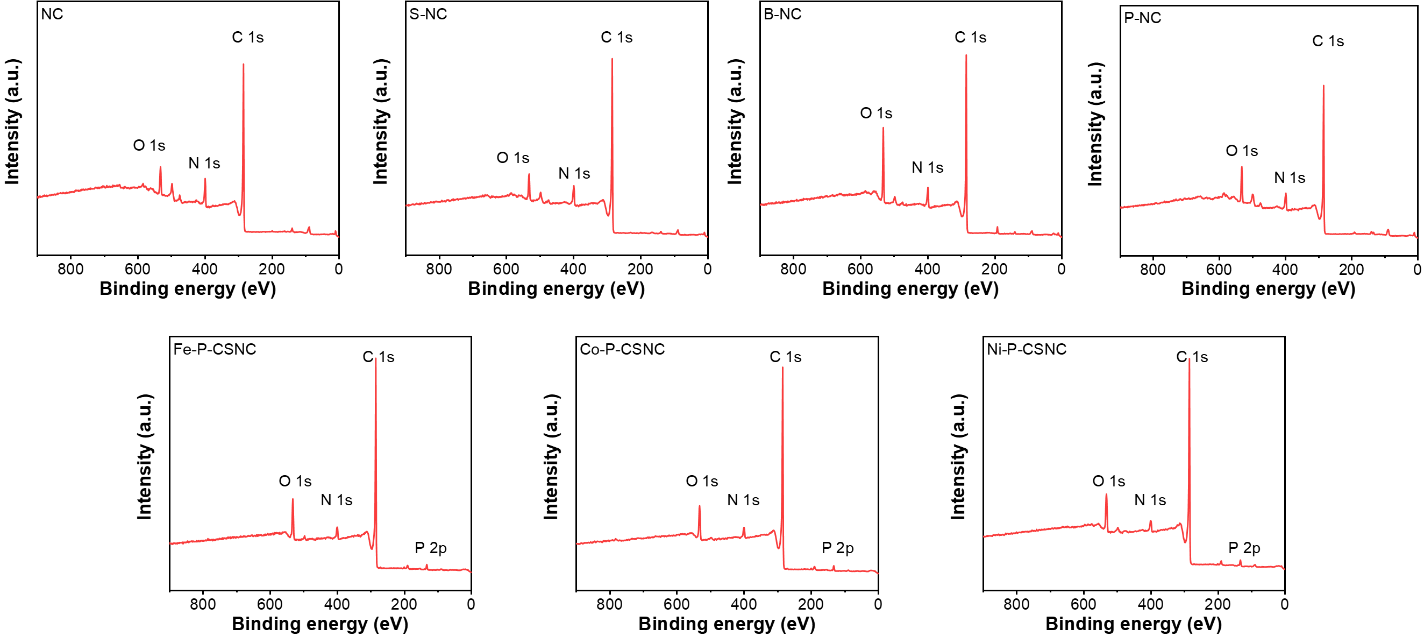


## Fig. S5 Full XPS spectra of different samples.


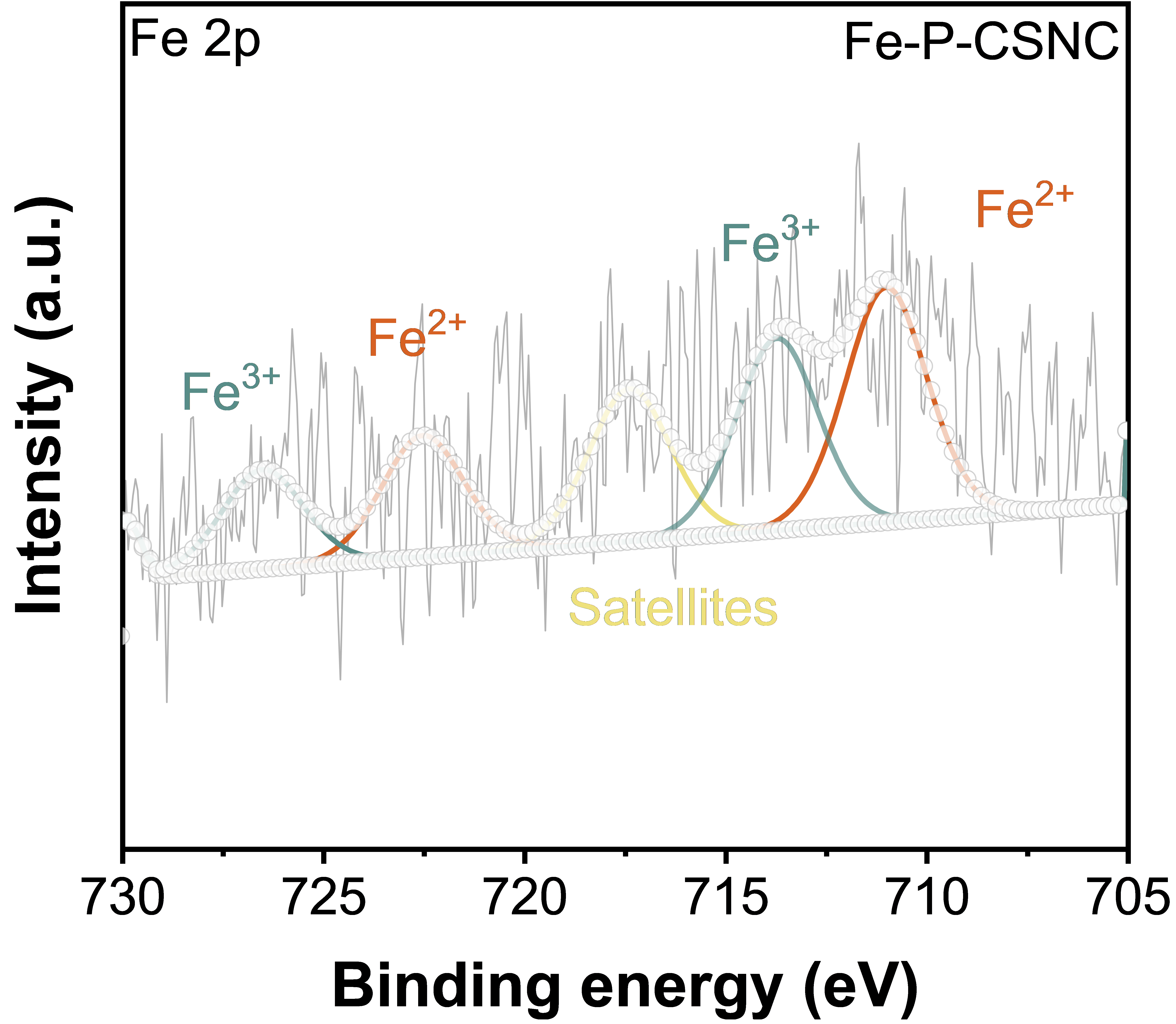


**Fig. S6** XPS Fe 2p spectra of Fe-P-CSNC.


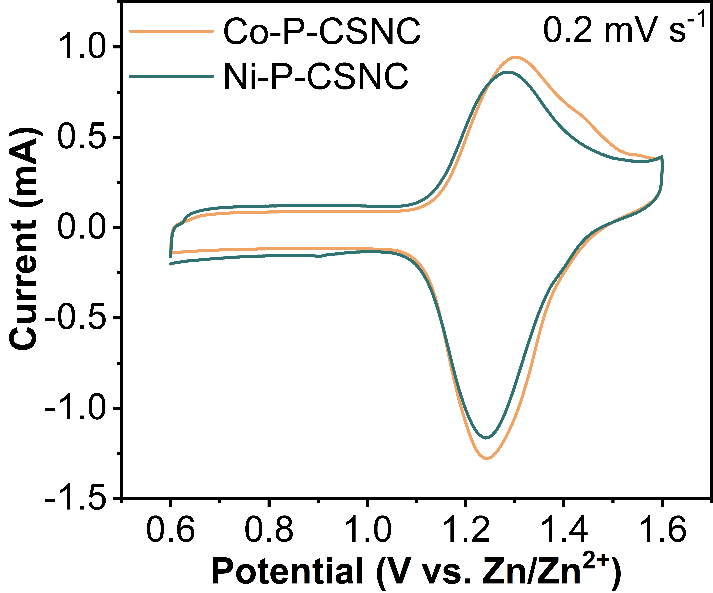


**Fig. S7** CV curves at 0.2 mV s^-1^.


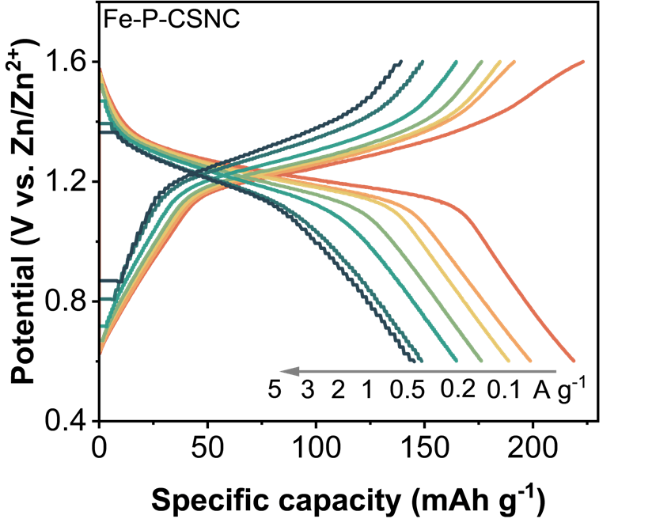


**Fig. S8** GCD curves of Fe-P-CSNC at different rates.


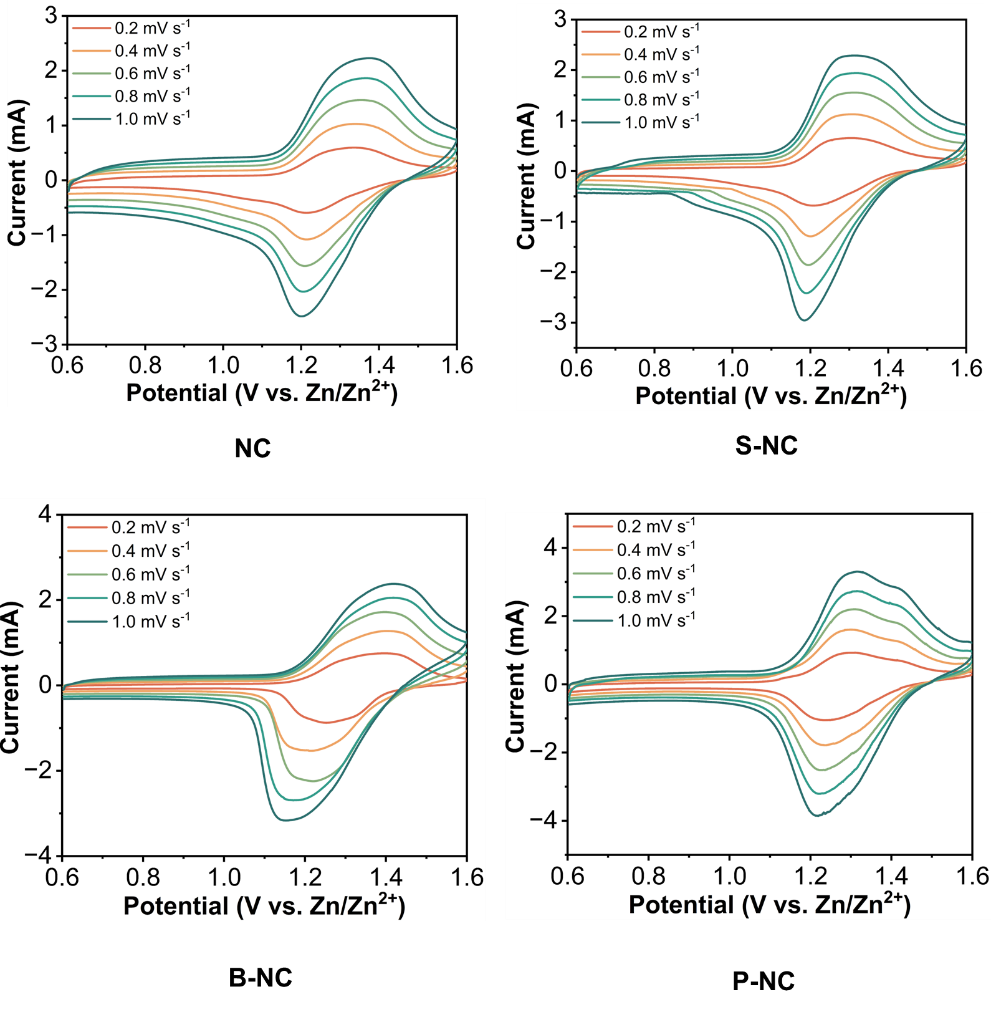


**Fig. S9** CV curves of M_1_-NC at different scan rate.


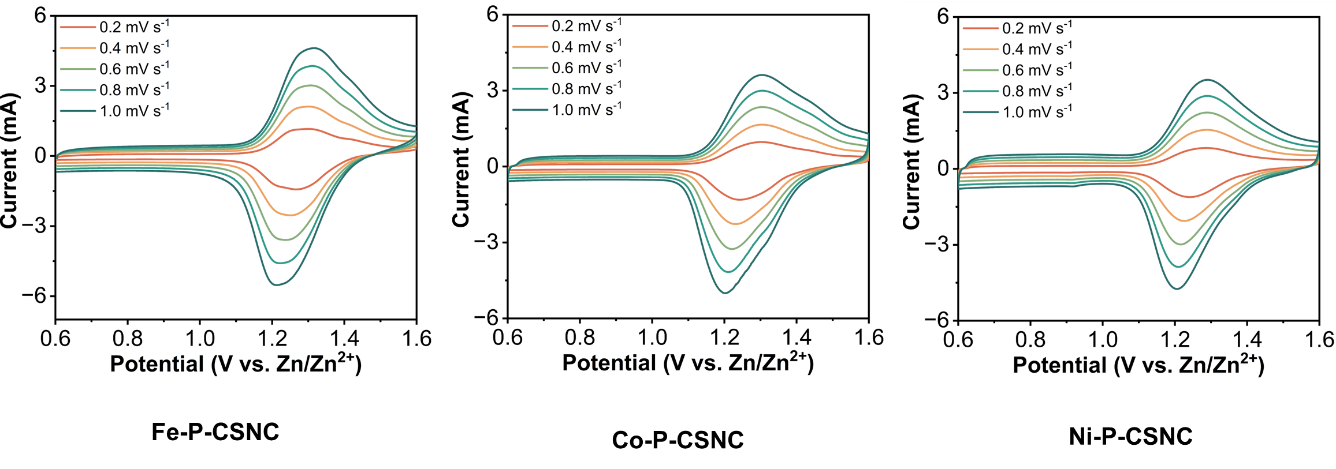
**Fig. S10** CV curves of M_2_-P-CSNC at different scan rate.


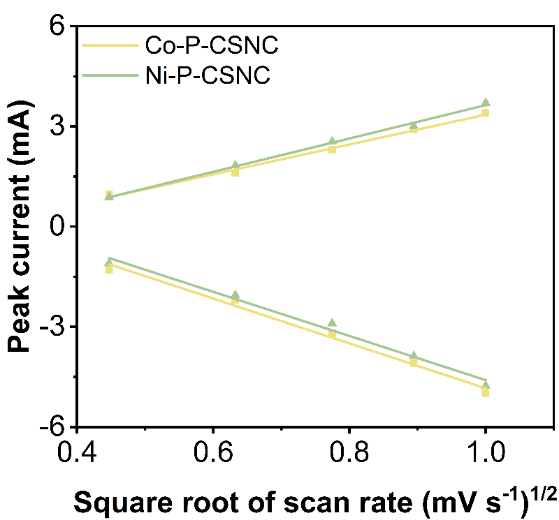


## Fig. S11 Linear fits of peak current with the square root of scanning speeds ranging from 0.2 to 1 mV s^−1^.


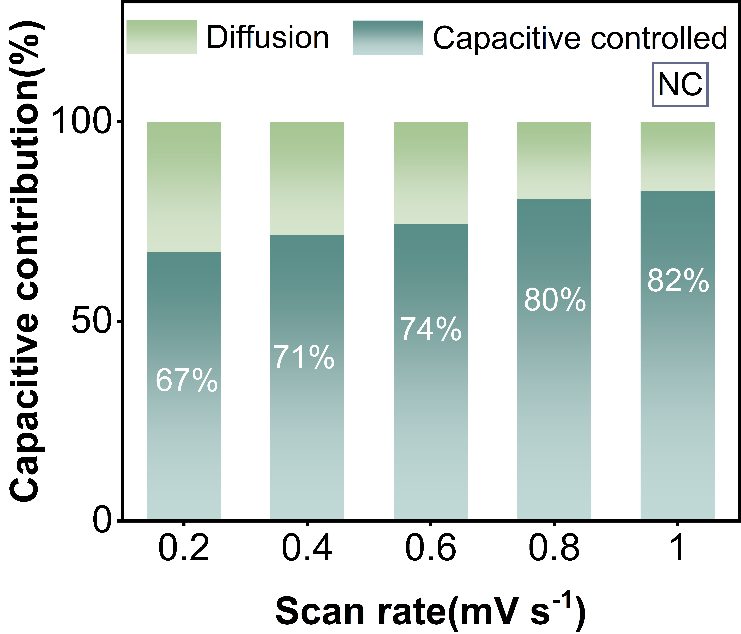


**Fig. S12** The capacitive contribution of NC at different scan rate.


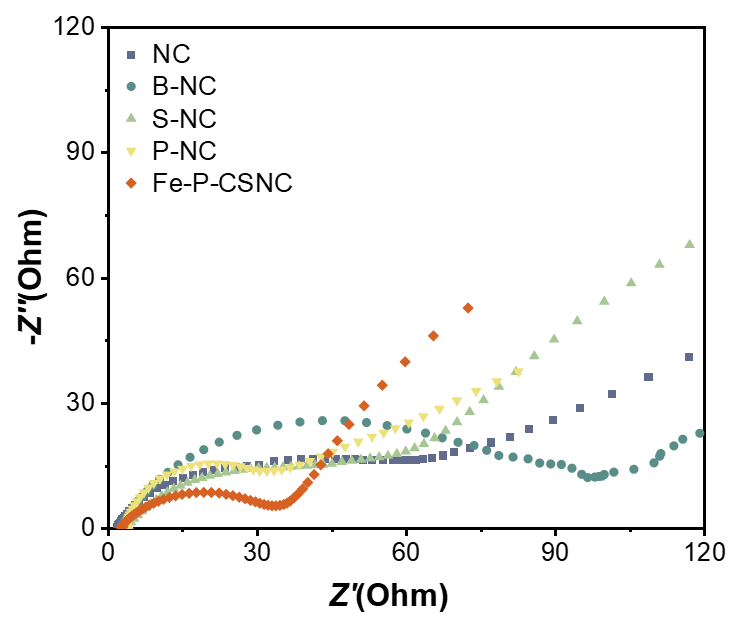


**Fig. S13** EIS curves of all samples.


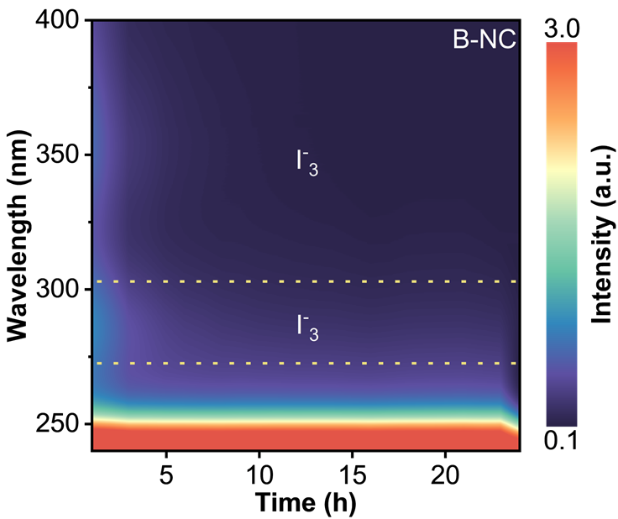


**Fig. S14** In-situ monitoring of polyiodide solution adsorption on B-NC.


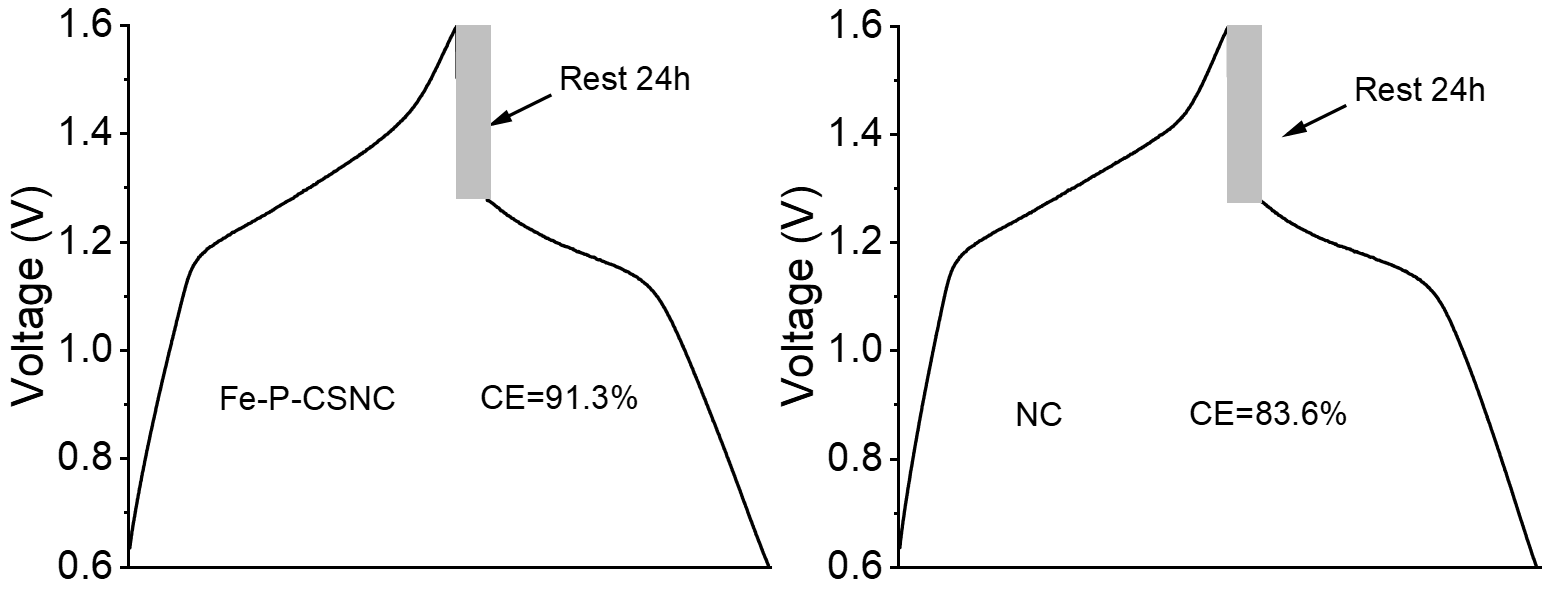


**Fig. S15** The self-discharge capacity of the two typical cathodes after 24 h.


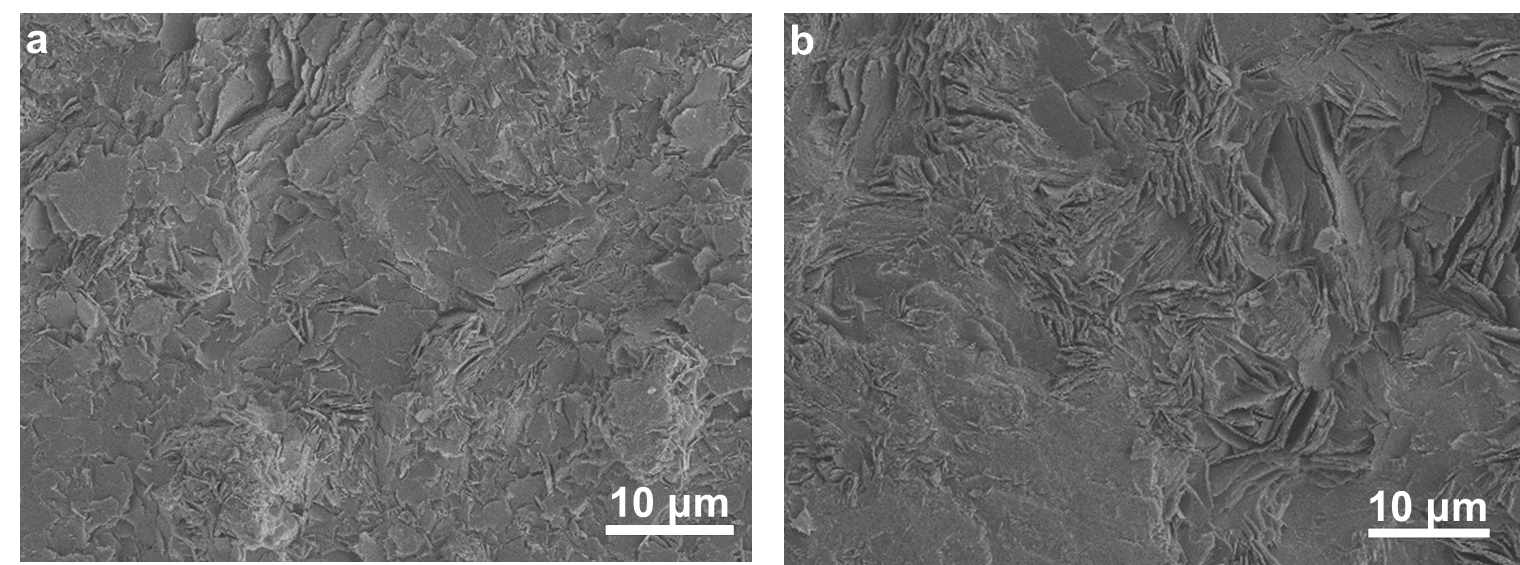


**Fig. S16** SEM images of Zn, **a** Fe-P-CSNC as cathode and **b** NC as cathode**.**


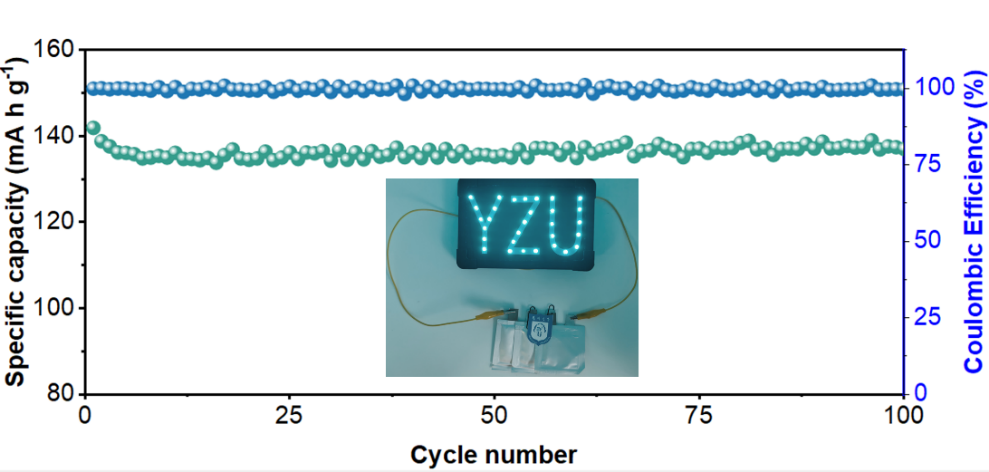


## Fig. S17 The cycle performance of pouch cells based on Fe-P-CSNC.


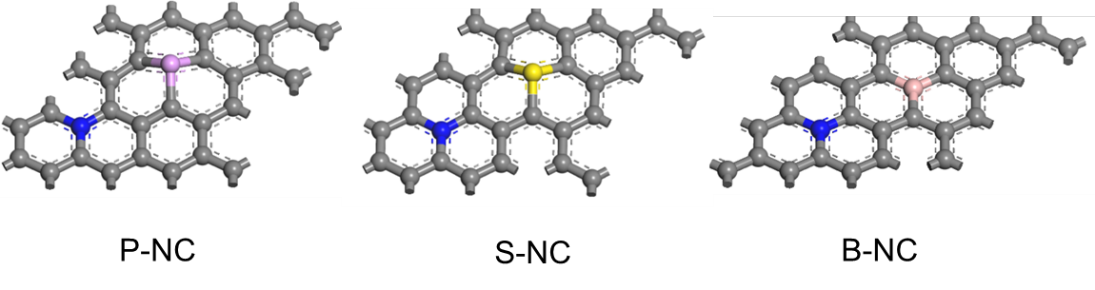


## Fig. S18 The optimized geometry conformation models (M_1_-NC).


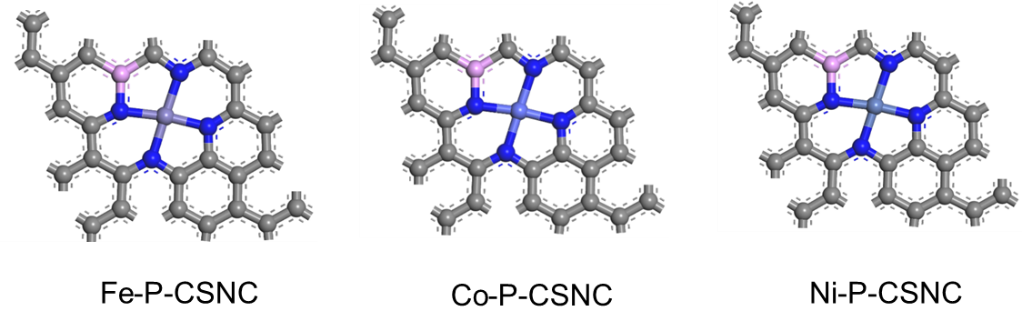


**Fig. S19** The optimized geometry conformation models (M_2_-P-CSNC).


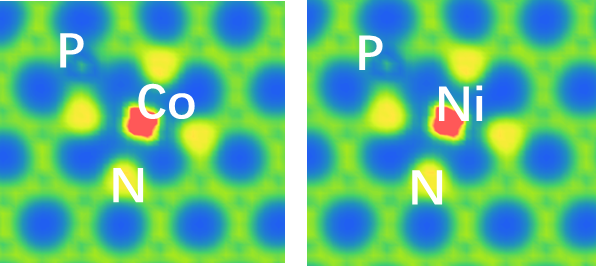


**Fig. S20** The charge distribution of Co-P-CSNC and Ni-P-CSNC.


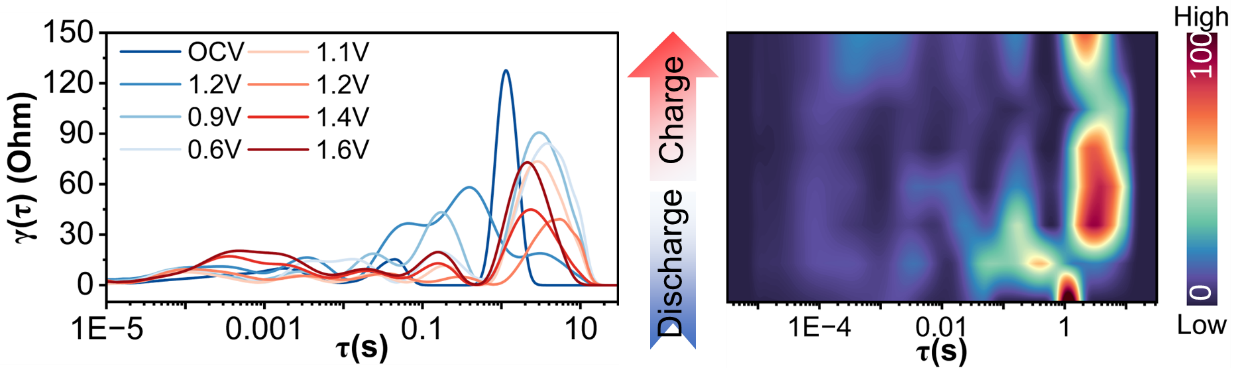


## Fig. S21 DRT calculated from EIS measurements of NC at different voltages and corresponding contour plots.

**Table S1** Elemental content of Fe element in Fe-P-CSNC measured by ICP-MS

| **Sample** | **Fe wt%** |
| --- | --- |
| Fe-P-CSNC | 1.96 |

**Table S2** Comparison of the Fe-P-CSNC/I_2_ electrode with reported aqueous Zn–I_2_ batteries

| Sample | Capacity/mA h g^–1^ | Cycles | Current density/mA g^–1^ | References |
| --- | --- | --- | --- | --- |
| NiSAs-HPC/I_2_ | 179 | 1000 | 1100 | [S1] |
| Fe-NCF/I_2_ | 169 | 1500 | 442 | [S2] |
| MPC/I_2_ | 112 | 2000 | 1000 | [S3] |
| PANI-I_2_ | 160 | 700 | 1500 | [S4] |
| PTCC900@I_2_ | 100 | 3000 | 500 | [S5] |
| B-Fe-NC/I_2_ | 158 | 10000 | 2210 | [S6] |
| NCCs/I_2_ | 259 | 1000 | 100 | [S7] |
| PNC-1000-I_2_ | 200 | 10000 | 1000 | [S8] |
| ZPC/I_2_ | 99 | 1000 | 800 | [S9] |
| ODAI_2_ | ~150 | 3500 | 1000 | [S10] |
| Fe-P-CSNC/I_2_ | 163 | 20000 | 2000 | This work |

**Supplementary References**

1. L. Ma, Y. Ying, S. Chen, Z. Chen, H. Li et al., Electrocatalytic selenium redox reaction for high-mass-loading zinc-selenium batteries with improved kinetics and selenium utilization. Adv. Energy Mater. **12**(26), 2201322 (2022). <https://doi.org/10.1002/aenm.202201322>
2. S. Ding, Q. Chen, S. Chen, Y. Tian, J. Zhang, The dispersion of iron nitride among porous carbon fibers to enhance redox conversion for high-performance zinc-iodine batteries. Chin. Chemical Lett. **34**(11), 108232 (2023). <https://doi.org/10.1016/j.cclet.2023.108232>
3. Y. Hou, F. Kong, Z. Wang, M. Ren, C. Qiao et al., High performance rechargeable aqueous zinc-iodine batteries *via* a double iodine species fixation strategy with mesoporous carbon and modified separator. J. Colloid Interface Sci. **629**, 279–287 (2023). <https://doi.org/10.1016/j.jcis.2022.09.079>
4. X. Zeng, X. Meng, W. Jiang, J. Liu, M. Ling et al., Anchoring polyiodide to conductive polymers as cathode for high-performance aqueous zinc–iodine batteries. ACS Sustainable Chem. Eng. **8**(38), 14280–14285 (2020). <https://doi.org/10.1021/acssuschemeng.0c05283>
5. Y. Wu, Y. Qian, C. Huang, Y. Zhang, Y. Yang et al., A high-performance aqueous Zn-I_2_ battery with polyacrylamide hydrogel electrolyte. Electrochim. Acta **460**, 142593 (2023). <https://doi.org/10.1016/j.electacta.2023.142593>
6. M. Liu, Q. Chen, X. Cao, D. Tan, J. Ma et al., Room temperature construction of vicinal amino alcohols via electroreductive cross-coupling of N-heteroarenes with aldehydes. J. Am. Chem. Soc. **144**, 21683 (2022). <https://doi.org/10.1021/jacs.2c11582>
7. W. Liu, P. Liu, Y. Lyu, J. Wen, R. Hao et al., Advanced Zn-I_2_ battery with excellent cycling stability and good rate performance by a multifunctional iodine host. ACS Appl. Mater. Interfaces **14**(7), 8955–8962 (2022). <https://doi.org/10.1021/acsami.1c21026>
8. T. Liu, H. Wang, C. Lei, Y. Mao, H. Wang et al., Recognition of the catalytic activities of graphitic N for zinc-iodine batteries. Energy Storage Mater. **53**, 544–551 (2022). <https://doi.org/10.1016/j.ensm.2022.09.028>
9. J. Xu, J. Wang, L. Ge, J. Sun, W. Ma et al., ZIF-8 derived porous carbon to mitigate shuttle effect for high performance aqueous zinc–iodine batteries. J. Colloid Interface Sci. **610**, 98–105 (2022). <https://doi.org/10.1016/j.jcis.2021.12.043>
10. X. Li, S. Wang, T. Wang, Z. Duan, Z. Huang et al., Flexible zinc-iodine batteries with ultralong cycle life and high volumetric energy density. Nano Energy **98**, 107287 (2022). <https://doi.org/10.1016/j.nanoen.2022.107287>
